# Identification of Functional Genetic Variations Underlying Flooding Tolerance in Brazilian Soybean Genotypes

**DOI:** 10.3390/ijms231810611

**Published:** 2022-09-13

**Authors:** Luisa Abruzzi de Oliveira-Busatto, Cecilia Paz da Silva Giordano, Marília Ferreira da Silva, Darci Uhry Junior, Frank Guzman, Beatriz Wiebke-Strohm, Zenglu Li, Christian Bredemeier, Maria Helena Bodanese-Zanettini

**Affiliations:** 1 Programa de Pós-Graduação em Genética e Biologia Molecular, Instituto Nacional de Ciência e Tecnologia: Biotec Seca-Pragas, Departamento de Genética, Instituto de Biociências, Universidade Federal do Rio Grande do Sul (UFRGS), Porto Alegre 91501-970, Brazil; 2 Programa de Pós-Graduação em Fitotecnia, Departamento de Plantas de Lavoura, Faculdade de Agronomia, Universidade Federal do Rio Grande do Sul (UFRGS), Porto Alegre 91540-000, Brazil; 3 Instituto Rio Grandense do Arroz (IRGA), Porto Alegre 90220-007, Brazil; 4 Escuela de Medicina, Universidad Científica del Sur, Lima 15067, Peru; 5 Department of Crop and Soil Sciences, University of Georgia, Athens, GA 30602, USA

**Keywords:** molecular markers, RNA-Seq, SNPs, abiotic stress, phenotyping

## Abstract

Flooding is a frequent environmental stress that reduces soybean (*Glycine max*) growth and grain yield in many producing areas in the world, such as, e.g., in the United States, Southeast Asia and Southern Brazil. In these regions, soybean is frequently cultivated in lowland areas by rotating with rice (*Oryza sativa*), which provides numerous technical, economic and environmental benefits. Given these realities, this work aimed to characterize physiological responses, identify genes differentially expressed under flooding stress in Brazilian soybean genotypes with contrasting flooding tolerance, and select SNPs with potential use for marker-assisted selection. Soybean cultivars TECIRGA 6070 (flooding tolerant) and FUNDACEP 62 (flooding sensitive) were grown up to the V6 growth stage and then flooding stress was imposed. Total RNA was extracted from leaves 24 h after the stress was imposed and sequenced. In total, 421 induced and 291 repressed genes were identified in both genotypes. TECIRGA 6070 presented 284 and 460 genes up- and down-regulated, respectively, under flooding conditions. Of those, 100 and 148 genes were exclusively up- and down-regulated, respectively, in the tolerant genotype. Based on the RNA sequencing data, SNPs in differentially expressed genes in response to flooding stress were identified. Finally, 38 SNPs, located in genes with functional annotation for response to abiotic stresses, were found in TECIRGA 6070 and absent in FUNDACEP 62. To validate them, 22 SNPs were selected for designing KASP assays that were used to genotype a panel of 11 contrasting genotypes with known phenotypes. In addition, the phenotypic and grain yield impacts were analyzed in four field experiments using a panel of 166 Brazilian soybean genotypes. Five SNPs possibly related to flooding tolerance in Brazilian soybean genotypes were identified. The information generated from this research will be useful to develop soybean genotypes adapted to poorly drained soils or areas subject to flooding.

## 1. Introduction

Flooding is a frequent environmental stress that reduces soybean (*Glycine max*) growth and grain yield potential in many producing areas in the world, such as, e.g., in the United States, especially the Mississippi delta region, Southeast Asia and Southern Brazil [1,2,3,4,5]. In these regions, soybean is frequently cultivated in lowland areas by rotating with rice (*Oryza sativa*) to provide numerous technical, economic and environmental benefits [6]. In this scenario, crop rotation with soybean is one of the most promising tools for weed control, especially weedy red rice, the most serious and frequent biotic limitation for cultivated rice [7,8]. Furthermore, the cultivation of soybean in lowland areas can improve soil fertility over time due to soybean’s biological nitrogen fixation and increase economical return to farmers because of the increase in world demand for plant-based protein sources. However, lowland soils typically have poor natural drainage and are subjected to temporal flooding, especially after heavy rain events, which entails a series of changes in soil–plant dynamics [9]. According to the Intergovernmental Panel on Climate Change (http://www.ipcc.ch, accessed on 12 December 2021), such events may become more frequent and intense in the future due to climate changes caused by anthropogenic action. As with the majority of grain crops, soybean is sensitive to flooding [10]. This species has wide genetic variability in relation to flooding tolerance, since it originated from areas subject to flooding in China [11,12].

Flooding stress occurs by the quick depletion of soil oxygen (O_2_). Dissolved oxygen has a diffusion of 10,000 times lower in water than in well-drained soils, which hinders gas exchange [13,14,15]. Cellular O_2_ can decline to concentrations that restrict aerobic respiration due to energy exhaustion [16,17,18,19]. In an attempt to maintain energy balance, the major land plants enhance the fermentative and glycolytic routes [1,9,20,21]. The key enzymes involved in the establishment of fermentative metabolism in plants during oxygen shortage are alcohol dehydrogenase (ADH) and pyruvate decarboxylase (PDC) [22,23]. 

Phytohormones also play a central role in the flooding tolerance mechanism [24,25,26]. For instance, the group VII ethylene response factor (ERF), highly preserved in plants, has been shown to be involved in modulating the response to flooding [27,28,29,30,31], indicating that the presence of ethylene can help soybean plants withstand this stress. In rice, ERF transcription factor genes *SUBMERGENCE 1A (SUB1A)*, *SNORKEL1 (SK1)* and *SNORKEL2 (SK2)* regulate contrasting strategies for plant survival under this stress. The *SUB1A* gene confers tolerance to complete submergence through the repression of cell elongation and carbohydrate metabolism, in addition to increasing fermentative metabolism activity by up-regulating the ADH enzyme [32,33], while *SK1* and *SK2* genes promote stem internode elongation in rice in response to deep water layers [34,35].

In Arabidopsis, five members of the ERF group VII (*HRE1, HRE2, RAP2.2, RAP2.12* and *RAP2.3)* have shown enhanced responses to low O_2_ [30,36,37]. The transcription factor *RAP2.2* is constitutively expressed at a higher level in roots than in shoots, but shows regulation induced in shoots by ethylene, and acts in signal transduction pathways mediated by this hormone [38]. The over-expression of *RAP2.2* increases the survival of seedlings under low O_2_, while mutants knocked out for this gene have a poor response to this stress [37], in addition to regulating the metabolic processes central to plant growth, development and resistance [39]. It has also been observed in stems that overexpressing *RAP2.12* induces a significant increase in the expression of hypoxia-responsive genes [40]. Studies with group VII ERFs have shown that they are stabilized under low O_2_ but targeted for proteolysis under normoxia [41,42,43,44], suggesting that ethylene and the turnover of ERF group VII, together with oxygen-dependent signal transduction, play a crucial role in stress response to hypoxia [27,29,45,46].

This research aims to characterize physiological and grain production responses and identify genes differently expressed under flooding stress in two soybean genotypes with contrasting flooding tolerance. In addition, we identify and test potential molecular markers (SNPs) associated with flooding tolerance. This information will help develop soybean cultivars adapted to poorly drained soils or areas subject to flooding.

## 2. Results and Discussion

### 2.1. Analysis of RNA-seq Experiment

RNA-seq libraries were sequenced and 47.9 to 48.3 million reads per lane were generated. A total of 81.8–82.3% of the total Illumina reads aligned to the *Glycine max* Wm82.a2.v1 [47] reference genome (Table 1). 

The expression of 53.2 to 53.6 thousand genes was investigated in this RNA sequencing analysis. An FDR ≤ 0.001 and the absolute value of log_2_ ratio ≥1 were used as the threshold to judge the significance of gene expression difference. According to the statistical criteria, we identified genes from the expression profile data with expression levels that were statistically altered by flooding stress. Comparing genotypes, TECIRGA 6070 presented 1494 and 731 genes up- and down-regulated, respectively, in the control condition. Regarding the flooding treatment, 752 and 871 genes were up- and down-regulated, respectively, in the tolerant genotype (Table 2).

**Figure 1:**
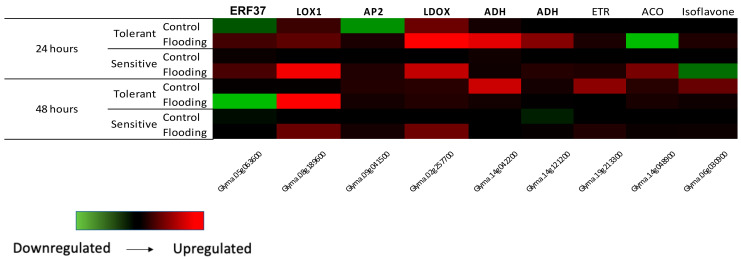
Real-time validation of RNA-Seq results.

RNA-seq data were validated using qPCR. ERF15, Isoflavone, LOX1, LDOX and two ADH genes were up-regulated in the tolerant cultivar (TECIRGA 6070) 24 h after flooding stress (Figure 1). In Arabidopsis and soybean, lipoxygenases are involved in increasing stress resistance and boosting defense reactions [48,49]. Under flooding stress, most soybean lipoxygenases are decreased in the roots [50]. Komatsu et al. (2010) [51] reported that two lipoxygenases were decreased in soybean in response to flooding stress and suggested that these enzymes affected cell wall metabolism due to the suppression of lignification. Taken together, the data indicate that lipoxygenase may regulate hormone pathways for stress tolerance and is involved in defense reactions in soybean under flooding stress. In our work, a lipoxygenase was found up-regulated in the tolerant cultivar. This gene could be involved in the tolerance mechanism.

Anthocyanins, as one of the most important water-soluble pigments in plants, have a wide range of biological functions, such as plant abiotic stress adaptation, defense against pathogen invasion, antioxidation and other health-related functions. Anthocyanins are flavonoids, and their biosynthesis and accumulation are parts of the flavonoid biosynthetic pathway. Leucoanthocyanidin dioxygenase (LDOX) is an enzyme present in this pathway and is responsible to form anthocyanidins, later converted into anthocyanins. Ding et al. (2022) [52] showed that the increased anthocyanin content could be related to strong waterlogging resistance in rapeseed (*Brassica napus*).

Transcription factors (TFs) representing basic helix–loop–helix (bHLH), ethylene response factors (ERFs), myeloblastosis (MYB), no apical meristem (NAC), and WRKY amino acid motif (WRKY) types are major families known to be involved in the mechanisms of stress tolerance.

The AP2/ERF transcription factor superfamily has been identified in many species and is divided into different families based on the number of AP2 domains, including the AP2, ERF, DREB, and RAV families. Ethylene accumulates rapidly under flooding conditions. The production of ethylene increased in waterlogged soybeans, with a significantly greater increase in tolerant lines than in sensitive lines [53]. The locus Glyma.05G063600 encodes an ethylene-responsive transcription factor 15-related, now named GmERF37, and belongs to Group I [54]. Chen et al. (2016) [55] identified 38 soybean ERFs up-regulated in leaf tissue under flooding stress. The authors observed that GmERF37 was up-regulated under flooding and drought stress. This suggests the role of ethylene in both drought and flood-responsive pathways. 

Regarding isoflavones, Coutinho et al. (2018) [56] observed that the amount of isoflavones in soybean genotypes decreased under flooding conditions, but highlighted that the mechanism of the down-regulation of isoflavones in soybeans under flooding stress remains unknown. On the other hand, it is suggested that soybean isoflavone levels are increased when plants are grown in areas subject to intermittent flooding [57]. In the present study, the increase was observed in the tolerant cultivar after 24 h of flooding. This could be a mechanism for plant tolerance to stress. Regarding alcohol dehydrogenase (ADH), the gene was also induced after 24 h of stress. 

Gene Ontology (GO) analyses were performed and DEGs were linked with their pathway (putative biological roles). Among the DEGs found in the tolerant genotypes at control and flooding conditions, 1712 presented pathway annotation. The most representative pathway was related to metabolic pathways (300 genes—17.5%), followed by ribosomes (191 genes—11.2%), the biosynthesis of secondary metabolites (183 genes—10.69%), plant–pathogen interactions (144 genes—8.41%), and plant hormone signal transduction (125 genes—7.3%) (Supplementary Figure S1). 

Ribosomal proteins are essential for protein synthesis, being involved in developmental processes, and act as environmental sensors [58]. They are commonly decreased in plants in response to abiotic stresses such as flooding [59], ultraviolet-B [60], and cold [61], retarding growth and impairing productivity [62]. In soybean, proteins related to protein synthesis were also identified in the roots of 2-day-old plants exposed to flooding under light or dark conditions. The 60S acidic ribosomal protein (Glyma12g30800.3 and Glyma03g35080.1) was down-regulated in both conditions (light and dark). However, the ribosomal protein S5/elongation factor G/III/V (Glyma08g18110.1 and Glyma15g40860.1) was up-regulated under light conditions [48]. Another study performed with soybean analyzed the rough ER fraction isolated from the root tips of 2-day-old plants submitted to flooding for two days and verified that 65% of ribosomal proteins identified were decreased in response to stress [63]. In the present work, many ribosomal proteins (60S ribosomal protein L3-like, 40S ribosomal protein SA, ribosomal protein S6, 40S ribosomal protein S11, 40S ribosomal protein S2, ribosomal protein S27, and others listed in Supplementary Table S3) were up-regulated in the tolerant cultivar (TECIRGA 6070) under flooding conditions compared with control conditions. Protein synthesis has previously been shown to be impaired in soybean roots under flooding, and it might be due to changes in ribosomal protein abundance [18,63]. Sharmin et al. [64] performed a protein–protein interaction analysis and identified three hub genes that could be associated with enhanced soybean seed-flooding tolerance: Glyma.01G207700 (ribosomal protein L23/L15e family protein), Glyma.05G016800 (ribosomal protein L23/L15e family protein) and Glyma.08G159800 (40 s ribosomal protein SA B). 

Stress signaling events comprise the production of secondary messengers, variation in the intracellular calcium concentration, and the activation of kinase cascades [65]. The endoplasmic reticulum is the organelle involved in calcium homeostasis and it is crucial for protein quality [66]. The increase in intracellular Ca^2+^ levels in response to stress conditions is perceived by calcium-binding proteins [67]. These proteins can bind to the promoter region of genes involved in the stress response or interact with DNA-binding proteins that regulate these genes, occasioning in gene activation or suppression [65]. Calcium encrypts the *stimuli* in calcium signatures, which among other sensors are also detected by calmodulin (CaM) protein [68]. The cytosolic calcium content has been found to be increased in soybean after flooding and drought stresses [63]. Here, two calmodulin-binding proteins (Glyma07g30990.1 and Glyma08g12100.1), a calmodulin-binding receptor (Glyma17g11810.2) and a calcium-binding protein (Glyma09g40740.1) were detected as up-regulated under flooding conditions in the tolerant genotype (Supplementary Table S3). Similarly, Sharmin et al. [64] detected 24 Ca^2+^ signaling-related up-regulated DEGs in a flood-tolerant wild soybean genotype relative to the sensitive genotype. Comparisons in soybean gene expression after 6 and 12 h flooding revealed that genes involved in the calcium signaling were significantly up-regulated after 12 h [69]. A previous study based on protein data reported that calmodulin-binding proteins were decreased in soybean root tips under flooding, whereas calcium-transporting ATPases were increased in response to this stress [63]. The contradictory response could be accounted to differences in genotypes, developmental stages (2–4-day-old vs. V6-growth-stage plants), analyzed tissue (cotyledon vs. leaves) and tolerance to stress. On the other hand, in the present work, several members of calnexin and calreticulin were also down-regulated in the tolerant genotype. These proteins are chaperones that fold newly synthesized polypeptides and modulate the calcium transport in the endoplasmic reticulum. In the same way, calreticulin and calnexin proteins were found to be differentially abundant in the root tips of soybean submitted to water stress (flooding and drought) [63]. Nanjo et al. [69] also observed altered levels of calnexin in soybean under flooding stress. Taken together, these results demonstrate that the protein folding process might be affected by water stress. 

To enable the coordination of genes and their regulators involved in stress remediation, there is a crosstalk among different plant hormones [70]. Nine groups of plant hormones were described to participate in the defense response and signaling pathways for an efficient reaction to environmental stresses [65]. Abscisic acid (ABA), ehtylene, jasmonic acid (JA) and salicylic acid (SA) are known to perform major roles in intermediating plant defense response against biotic and abiotic stresses [71]. The expression of some regulatory genes can significantly affect stress tolerance due to their role in enhancing stress signals to regulate downstream responsive genes [72]. In this scenario, the transcription factors responsive to stress are important tools to manipulate the plant stress response [73]. Here, regulator molecules, such as transcription factors, phosphatases and kinases, were also differentially expressed when comparing control and flooding conditions. Among the stress-related transcription factors, several members of the NAC, APETALA2/ethylene-responsive factors (AP2/ERFs), bZIP, DOF, WRKY, GRAS and MYB proteins were detected. Members from the NAC, MYB and MYC families are known to function in an ABA-dependent manner. ERFs are the main downstream regulatory factors of ET signaling pathways in response to stress. DELLA proteins (members of the GRAS family of TF) function as integrators of the gibberellic acid (GA) and ABA signaling pathways [65,74].

Ten out of eleven WRKY genes were identified and up-regulated in the tolerant cultivar under flooding conditions (GmWRKY8, GmWRKY20, GmWRKY27, GmWRKY36, GmWRKY42, GmWRKY46, GmWRKY52, GmWRKY126 and GmWRKY149). The only WRKY gene down-regulated was GmWRKY144 (Supplementary Table S3). Similarly, previous studies have reported that the expression of WRKY-encoding genes is quickly and strongly induced in response to flooding in *A. thaliana*, and this is correlated with the induction of innate immunity marker genes [75,76,77]. Likewise, maize WRKY6 was induced in two sensitive genotypes more than two-fold at two time points after submergence (24 and 72 h) relative to the control samples. On the other hand, the expression of WRKY6 was down-regulated at both time points relative to controls in the two tolerant genotypes. Two others putative maize WRKY genes (similar to AtWRKY33) were down-regulated after submergence when compared to the sensitive genotypes [78]. Sharmin et al. [64] analyzed the transcriptome profile in roots of two contrasting wild soybean genotypes and verified that some transcription factors were uniquely expressed in the tolerant genotype. Among them are RADIALIS, Trihelix, and UNE, whereas E2F, EGL, GLK1, ORG, PIF, SWI/SNF and TGA were uniquely expressed in the sensitive genotype. The authors also suggest that three soybean GmERFVIIs (GmERFVII1-Glyma.02G016100; GmERFVII2-Glyma.09G041500 and GmERFVII3-Glyma.15G152000)) could play a key role in regulating seed-flooding tolerance in soybean, highlighting the importance of AP2/ERF transcription factors.

Nodulins are membrane channel proteins that facilitate the diffusion of water and small uncharged solutes which are involved in the symbiotic processes between legumes and rhizobia. Here, four nodulin-encoding genes (Glyma20g30580; Glyma04g34550; Glyma02g15380 and Glyma17g08110) were up-regulated under flooding stress (Supplementary Table S3). Similar results were obtained for *A. thaliana*. Under anaerobic conditions (such as, e.g., flooded soil), AtNIP2;1 was highly expressed in the root tips [79].

Concerning the contrasting genotypes present at stress conditions, 1032 DEGs have shown pathway annotation. Of those, 24.8% belong to the metabolic pathways (256 genes), 18.3% to the plant–pathogen interaction pathway (189 genes), 15.3% to the biosynthesis of secondary metabolites (158 genes), and 9.7% to plant hormone signal transduction (100 genes) (Supplementary Figure S2). The organic substance metabolic process and primary metabolic process were the most abundant categories in the GO process, comprising 47.0 and 44.3% of the sequences, respectively. Those compounds from primary metabolic processes are formed as a part of the normal anabolic and catabolic processes and are necessary to generate biomass precursors.

SNF-1, a SnRK1 (sucrose non-fermenting-1-related protein kinase 1) gene, was up-regulated in the tolerant genotype TECIRGA 6070 under flooding conditions (Supplementary Table S4). The SnRK1-A pathway induces MYBS1, which activates the RAmy3D starvation-inducible α-amylase gene [80]. SnRK1 is a central integrator of energy-related signals to coordinate starvation responses in plants, and it is inhibited by T6P (trehalose-6-P-phosphate). In rice, the locus OsTPP7, which encodes a T6P, has been identified as responsible for anaerobic germination tolerance, which enables uniform germination and seedling establishment under submergence [81]. Sucrose in high levels will result in higher levels of T6P and, therefore, the repression of SnrK1 and the down-regulation of α-amylases [2].

Considering the up-regulated genes in the tolerant genotype under control conditions, the highest differential expression was detected for ferritin (Glyma07g19060.1 and Glyma01g31300.1), Snakin-1 (Glyma06g04740.1), lectin precursor (Glyma02g18090.1), P21 protein (Glyma05g38130.1) and ribosomal proteins (Supplementary Table S2).

### 2.2. Physiological Variables

Chlorophyll fluorescence assessments were performed three times: immediately before the onset of flooding (time 0), during the flooding period (24 and 48 h of flooding) and after tank drainage (24 and 48 h of recovery). The PSII quantum yield evaluated in the period before flooding did not show any difference between the genotypes and this behavior remained constant in the plants maintained in the control condition (Supplementary Figure S3). Among the plants subjected to stress with 24 h of flooding, the sensitive genotype (FUNDACEP 62) showed significantly lower PSII quantum yield compared to the tolerant one (TECIRGA 6070). After 48 h of flooding, there was no difference between the genotypes for this parameter, probably due to the stress intensity. In the evaluation carried out 24 h after drainage, FUNDACEP 62 again presented lower quantum yield compared to TECIRGA 6070. In the evaluation performed 48 h after drainage, the genotypes did not differ (Supplementary Figure S3).

The O_2_ consumption by roots, fauna and soil microorganisms can result in O_2_ soil depletion within 24 h after the beginning of flooding [82]. As a result, photosynthesis is inhibited. Chlorophyll fluorescence was as an efficient parameter to characterize and differentiate the magnitude of stress caused by excess water between the genotypes at two times: 24 h of flooding and 24 h after drainage. The sensitive genotype, FUNDACEP 62, showed a sharper drop in quantum yield when stressed and a slower recovery after tank drainage (Supplementary Figure S3).

The occurrence of unfavorable environmental conditions, such as flooding, can induce the excessive production of reactive oxygen species (ROS). In this study, the content of hydrogen peroxide (H_2_O_2_) and the activity of the ROS detoxifying enzyme ascorbate peroxidase (APX) were evaluated (Supplementary Figure S4). It was observed that in plants submitted to 24h of flooding, the H_2_O_2_ content increased in relation to the control in both genotypes. However, this increase was more pronounced for FUNDACEP 62 (sensitive) than for TECIRGA 6070 (tolerant). In the second evaluation carried out after 48h of flooding, despite decreasing the levels of H_2_O_2_ relative to control, FUNDACEP 62 still showed higher levels of hydrogen peroxide when compared to the control. For TECIRGA 6070, on the other hand, hydrogen peroxide content in plants submitted to 48 h of flooding did not differ from the control condition. In the evaluation carried out 24 h after the tanks were drained, the H_2_O_2_ content of the two cultivars did not differ from their respective controls (Supplementary Figure S4A). ROS are often associated with oxidative stress. However, studies have shown that ROS play an important role in stress signaling and perception in plants [83,84,85]. The results of the present study in soybean agree with the results found in Arabidopsis [27], indicating that in the tolerant genotype TECIRGA 6070, the production of H_2_O_2_ may act on the perception and signaling of flooding stress.

Regarding APX activity, FUNDACEP 62 showed an increase as a function of flooding for 24 h, whereas in the second evaluation (48 h of flooding), it did not differ between plants under stress and control conditions. Moreover, the genotype TECIRGA 6070 showed lower APX activity in response to flooding for 24 and 48 h compared to its control (Supplementary Figure S4B). In a study with two other Brazilian soybean genotypes (Embrapa 45 and BR4, considered tolerant and sensitive to flooding, respectively), the inhibition of APX enzyme activity was observed for Embrapa 45 (tolerant) in response to hypoxia [86]. These results indicate that in hypoxia-tolerant soybean cultivars, the activity of the APX enzyme is initially inhibited when submitted to flooding.

Regarding nitrogen (N), the lack of oxygen in the root system of the soybean plant impairs the nodulation and inhibits the biological N fixation by the symbiotic bacteria in previously formed nodules [87,88]. Consequently, the total nitrogen accumulated in the plant is a more sensitive parameter than the accumulation of biomass in the shoot when analyzing flooding responses [89]. Soybean plants subjected to flooding for 14 days showed a lower accumulation of dry mass in the roots and shoots [90]. In another study, reductions of up to 55% were observed in the accumulation of shoot dry mass in soybean plants subjected to flooding [57]. 

In the present study, N uptake in the shoots was evaluated at two times, namely, 48 h after tank drainage and at flowering. At 48 h after drainage, there was no significant difference in nitrogen uptake between the treatments (flooding and control) for both genotypes. On the other hand, at flowering, the sensitive genotype FUNDACEP 62 showed a marked decrease in N accumulated in shoots when compared to the control. The tolerant genotype TECIRGA 6070 showed the stability of N uptake, and no significant difference was observed between treatments for this genotype (Supplementary Figure S5).

### 2.3. SNP Discovery

The RNA sequencing database was used for mining SNPs, using the genotype Williams 82 as a “reference sequence”. SNPs with a minimum coverage of 20 reads in each library were identified. Transition SNPs were predominant in relation to transversion, and the variation C/T was more prevalent in all four libraries analyzed.

A final number of 3000 SNPs present in the tolerant genotype (TECIRGA 6070) and absent in the sensitive one (FUNDACEP 62) were selected. Only SNPs in genes with functional annotation to respond to abiotic stresses were filtered out, aiming to reduce the number of candidate genes, resulting in thirty-eight genes. To validate the polymorphism potential to be used as a molecular marker, SNPs were selected with allele frequencies lower than 40% from the Phytozome database. Thus, 22 SNPs in 17 candidate genes distributed on 11 chromosomes were identified and selected for validation (Table 3). Calmodulin (Glyma.03G00410.0), DNA J homolog (Glyma.12G19010.0) and chlorophyll a-b-binding chloroplastic (Glyma.13G28200.0) genes presented more than one polymorphic site. 

### 2.4. Genotyping of an RIL Population

The genotyping of 11 lines with contrasting flooding tolerance (Williams 82, TECIRGA 6070, NA 5959, BMX Valente, BMX Ponta, NA 5909, SYN 1359, Woodruff, Benning, Boogs and PI 416937) was performed using the 22 SNPs identified (Table 3). Eight SNPs were selected due to the fact that both tolerant cultivars, TECIRGA 6070 and Benning, had the same alleles. Genotyping 128 RILs derived from Benning × PI 416,937 indicated that two SNPs (GSM0612 and GSM0613) were significantly associated with visual scores of flooding tolerance and plant survival rate in 2014 and 2015 (Table 4). The fact that only 2 of 22 SNPs were validated may be related to the fact that the population tested comes from the crossing of North American genotypes. For this reason, the SNPs were tested in field experiments using a panel of Brazilian soybean genotypes.

### 2.5. Field Experiments and SNP Validation in the Brazilian Soybean Genotypes

To investigate the contribution of the identified polymorphisms to the flooding tolerance, the phenotypic and grain yield impacts were analyzed in a panel of 166 Brazilian soybean genotypes (Supplementary Table S2). The phenotyping score of the plants (visual damage scale) was performed after the flooding period in four field experiments conducted in two growing seasons.

In Experiment 1, the flooding stress was imposed for five days, and plant phenotyping was performed six days after water drainage. Nine SNPs (GSM0600, GSM0601, GSM0602, GSM0605, GSM0616, GSM0620, GSM0621, GSM0622, GSM0623, and GSM0625) were identified as possibly related to the flooding response (Table 5 and Supplementary Table S4). The markers that presented the lowest (7.31) and highest (8.24) mean visual phenotyping scores were GSM0621 (TT allele) and GSM0601 (AA allele), respectively. However, the TT allele (GSM0621) was not very representative within the sampled genotypes, being found in only 2 out of the 96 genotypes investigated in this experiment (Table 5).

Regarding the second experiment, flooding was imposed for five days, and three phenotyping studies (visual score evaluations) were performed (at 8, 23 and 43 days after water drainage). The first, second and third phenotyping evaluations presented five, five and four significant SNP markers associated with greater flooding tolerance, respectively (Table 5). The significant SNPs obtained in all phenotyping evaluations were: GSM0601, GSM0604, GSM0612 and GSM0613. The alleles associated with the highest tolerance were AA (GSM0601 and GSM0604), TT (GSM0612) and GG (GSM0613). The markers corresponding to the lowest (4.63 (second phenotyping)) and the highest (6.79 (first phenotyping)) mean value of the visual score were GSM0619 (GG allele) and GSM0600 (CC allele), respectively, with GSM0619 being significant in the second evaluation of visual score. However, the GG (marker GSM0619) and CC (GSM0600) genotypes were not very representative within the investigated genotypes, being found in only 2 and 3 genotypes, respectively, from 32 genotypes (Supplementary Table S5).

In Experiment 3, the phenotyping was extended for longer periods: up to 60 days after water drainage. One, three, two, and five SNPs were significant in the first, second, third, and fourth phenotyping evaluations, respectively (Table 6). The SNPs GSM0612 and GSM0613 were significantly associated with a better response to flooding in three of the four evaluations performed. The alleles related to the highest tolerance were TT (GSM0612) and GG (GSM0613). The markers that presented the lowest (5.32 (fourth phenotyping)) and the highest (6.29 (fourth phenotyping)) visual score mean were GSM0599 (GG allele) and GSM0611 (CC allele), respectively. However, the GG allele (GSM0599) was not very representative within the genotypes used in this experiment, being found in only 2 out of the 26 genotypes investigated (Table 6 and Supplementary Table S6).

In Experiment 4, only GSM0604 (TT allele) and GSM0605 (TT allele) were associated with greater tolerance to flooding (lower values on the visual assessment scale and significant *p*-value), with the GSM0605 marker being significant for the three evaluations performed (Table 6). According to this experiment, this marker proved to be efficient in discriminating the best response to flooding of the Brazilian soybean genotypes tested. The markers corresponding to the lowest (6.41 (first phenotyping)) and the highest (7.27 (third phenotyping)) visual score mean were, respectively, GSM0605 (TT allele) and GSM0604 (AA allele), which were both significant for visual score values (Table 6 and Supplementary Table S7).

Considering the 22 SNPs analyzed in all the field experiments, 17 had a significantly different phenotyping score among the contrasting genotypes (Table 5 and Table 6). Furthermore, the SNPs GSM0601, GSM0604, GSM0605, GSM0612, GS0M613, GSM0616 and GSM0625 were significantly related to flooding response in two different experiments. These results demonstrate the viability and efficiency of these SNPs in identifying differential responses of soybean genotypes to flooding. 

In order to better comprehend the tolerance of the Brazilian soybean genotypes to flooding, grain yield was also evaluated and correlated with SNP data (Table 5 and Table 6). In Experiment 2, six SNPs were significantly related to higher productivity under flooding: GSM0601 (AA allele), GSM0602 (TT allele), GSM0604 (AA allele), GSM0612 (TT allele), GSM0613 (GG allele) and GSM0625 (AA allele) (Table 5). In Experiment 3, only two SNPs presented an association with higher productivity: GSM0611 (TT allele) and GSM0625 (AA allele) (Table 7). The AA allele of the GSM0625 marker showed a significant relationship to higher grain yields in both experiments. The markers that presented groups with the highest yields of 1807 and 1214 kg ha^−1^ were GSM0601 (Experiment 2) and GSM0625 (Experiment 3), respectively. On the other hand, the lowest average grain yields were 1222.5 and 802 kg ha^−1^, obtained in sample groups of the markers GSM0602 (AA alleles) (Experiment 2) and GSM0600 (CC alleles) (Experiment 3), respectively (Supplementary Table S8).

To further explore the consequences of flooding on plant development and the validation of the molecular markers, evaluations of plant mortality, normalized difference vegetation index (NDVI), and leaf retention were carried out. Among the 22 analyzed SNPs, it was possible to observe an association in only two (GSM0605 and GSM0611) in Experiment 3 (Table 7). The GG allele (GSM0605) was associated with higher NDVI and higher leaf retention. Regarding the SNP GSM0611, the TT allele showed greater leaf retention. No significant relationship of the SNPs with plant mortality was observed.

On the other hand, in Experiment 4, the SNPs GSM0605 (TT allele), GSM0612 (TT allele), GSM0613 (GG allele) and GSM0614 (CC allele) were significantly associated with lower mortality of soybean plants. In this same experiment, GSM0604 (TT allele), GSM0605 (TT allele) and GSM0618 (TT allele) were significant for a higher NDVI (Table 7 and Supplementary Table S9). Based on the results of plant mortality and NDVI, the TT allele of the GSM0605 has potential to predict a greater tolerance to flooding.

**Figure 2:**
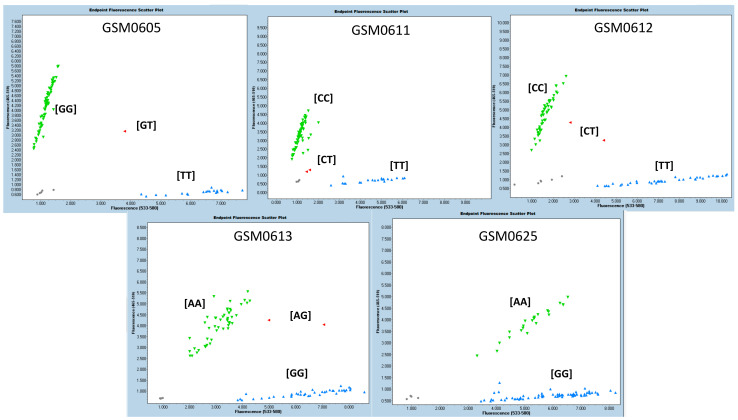
Genotyping plot of KASP assays for GSM0605, GSM0611, GSM0612, GSM0613 and GSM0625.

The SNPs that showed statistical significance in at least two characteristics/variables analyzed, or in two experiments, with allelic consistency that exhibited the best performance, were selected. According to the phenotyping (visual score) and grain yield evaluations, five molecular markers were selected as promising for use in assisted selection: GSM0605, GM06011 and GSM0612, GSM0613, and GSM0625 (Table 8). The methodology used (KASP) precisely grouped the three genotypic classes (two homozygous groups and one heterozygote group) based on these SNPs. In Figure 2, the graphs generated for the five previously selected markers are shown. The groupings of the three genotypic classes indicate, for each marker, that they can effectively differentiate the evaluated genotypes. The results showed that homozygous genotypes were more frequent than heterozygous ones in all cases. The genotypes with the presence of promising markers were selected through the joint analysis of the five recommended molecular markers. The alleles related to the best performance for the tags GSM0605, GSM0611, GSM0612, GSM0613 and GSM0625 were TT, TT, TT, GG and AA, respectively. Thus, the genotypes BRB 16-253244, HO TERERÊ, SYN 1451946, BRB 16-238251 and NS 6700 simultaneously presented the five alleles associated with greater flooding tolerance. The genotypes BRB 16-236559, TMG 7058, BRB 16-242992, PIO 95R95, BS 1691, DM 66I68, SYN 1561, TEC 7849, M 5838, M 6410, BMX Ponta, CZ 26B42 and M5947 showed four alleles associated with greater tolerance to flooding. The other genotypes analyzed had a maximum of three alleles (including TECIRGA 6070) associated with greater tolerance to flooding (Table 9).

**Figure 3:**
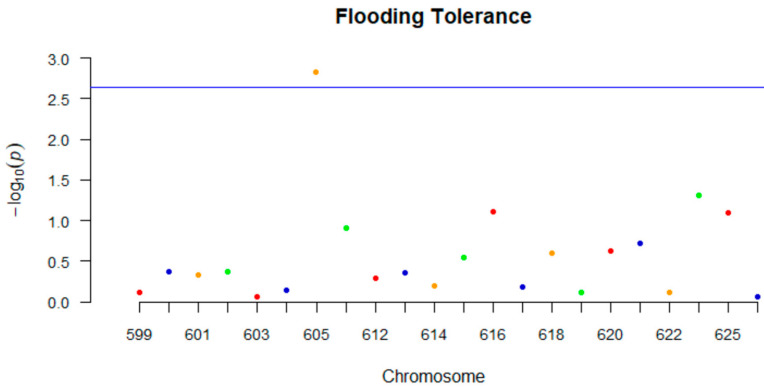
Manhattan plot of the results from the Mini-GWAS meta-analysis: The *y*-axis represents –log (two-sided *p* values) for association of variants with flooding tolerance from the meta-analysis. In the *x*-axis, the molecular marker codes (GSM0XXX) are represented. The horizontal blue line represents the threshold for genome-wide significance.

Regarding the GLM analysis, ANOVA indicated that effects of genotype, experiment and replicates were all significant and genotype *x* experiment was significant as well (*p* < 0.01). Mini-GWAS identified one marker (GSM605) on chromosome 9 that was significant for flooding tolerance (Figure 3). Single-factor analysis using both BLUPs and fixed effects identified three markers located on chromosomes 9, 10, and 13, respectively, which were significant (*p* < 0.05). Of these three markers, one marker was same as the one identified using Mini-GWAS (Table 10).

SNPs are the most abundant source of DNA polymorphism, with an estimated four to five million SNPs in the soybean genome. A study carried out with the main soybean varieties grown in the United States identified 280 SNPs in more than 76 Kbp of coding sequences [91]. However, the frequency of SNPs is relatively low in soybean compared to other cultivated crops. This characteristic is mainly due to the low genetic variability of the ancestor and the loss of variability during domestication [91,92,93].

Several studies have sought to understand the genetic and molecular mechanisms of tolerance to flooding in soybean. In this sense, several QTLs have been observed to be associated with greater tolerance to flooding. VanToai et al. [94] identified a QTL linked to Sat_064, located on chromosome 18, as associated with greater tolerance to flooding using two populations of recombinant strains (RILs). Cornelious et al. [95] reported five QTL markers associated with flooding tolerance as well. The Satt485 marker is located on chromosome 3, the Satt599 marker on chromosome 5, and three (Satt160, Satt269, and Satt252) markers are located on chromosome 13. They have been associated with flooding tolerance in two populations of recombinant strains. Githiri et al. [96] identified seven QTLs (ft1 to ft7) associated with grain yield under flooding stress, resulting in a proposed QTL close to Satt100.

Wang et al. [97] mapped three QTLs-Satt531-A941V (chromosome 1), Satt648-K418_2V (chromosome 5), and Satt038-Satt275 (chromosome 18) that were associated with greater tolerance to flooding in soybean. However, all these previously identified QTLs were discovered using linkage mapping resulting in a restricted mapping resolution due to limited recombination events in a biparental population. To overcome these limitations, high-resolution, high-throughput genomic analysis technologies have been developed, enabling new approaches for marker-assisted selection, such as genomic-wide association studies (GWAS). This type of study aims at genetic mapping through associations between loci and phenotypic characteristics in the population and seeks to detect the effects of genes on the genetic values of individuals [98].

Based on the above, Wu et al. [99] performed GWAS using a mapping panel composed of several introductions of soybean plants (PIs) obtained from the USDA germplasm bank to detect SNPs associated with greater tolerance to flooding. The genotypes were originally collected from 17 countries and 9 unknown origins, and were tested for two consecutive years in the field. The plant leaf damage score was used to index the soybean response to flooding stress. Fourteen SNPs were identified as being associated with greater tolerance and five SNPs were in candidate gene coding regions.

Flood-related QTL mapping studies show a wide distribution in the genome, indicating that greater soybean tolerance is a complex quantitative trait controlled by many genes with less effect [94,95,96,97,100,101].

Flooding induces gene expression and phenotypic alterations. Symptoms of flooding stress include leaf chlorosis, defoliation and plant death. The phenotypic variance observed among genotypes is partly due to genetic differences and partly to the difference in the environment, implying the possibility of changes in selection criteria depending on the environment [102]. Thus, it is important to understand the phenotypic variation that corresponds to the environment and the variation corresponding to the genotype to be able to estimate with better experimental precision the response of genotypes in different environments.

Phenotyping through visual score has been used to select genotypes with greater tolerance to flooding [103]. It is reported that flooding reduces soybean grain yield by 40 to 50% in genotypes with greater tolerance and by 70% to 80% in genotypes with greater sensitivity to this stress, varying according to the growth stage and duration of stress [95,103,104]. Thus, plants with greater tolerance to flooding are of enormous interest to farmers, as soybean is commonly sensitive to this stress condition [105].

Previous studies have assessed flooding tolerance in soybeans using field trial methods [103,106,107]. However, a small number of studies have identified QTLs or genes related to traits linked to grain yield. Hu et al. [108] phenotyped 113 wild soybean accessions related to productive traits, identifying 892 alleles and 18 markers for the evaluated traits. Two markers, namely, sct_010 and satt316, which are associated with grain yield, were expressed stably over two years at two experimental sites. Since these characteristics are complex and quantitative, environmental variations can trigger and modify the expression of genes related to them [109].

The five SNPs selected in the present study are distributed on chromosomes 3, 4, 9, and 13. Wu et al. [99] found SNPs associated with a greater tolerance to flooding on chromosomes 3, 4, 7, 13, and 19. Although chromosomes 3, 4, and 13 were found in both studies, no common locus is shared between them. In our study, the validated SNPs were in genes likely to be involved in the variation of tolerance to the flooding conditions. Wu et al. [99] found SNPs in candidate genes that perform diverse biological functions, including heat stress regulation, protein phosphorylation, DNA repair, histone methylation, and protein degradation.

Among the genes with SNPs associated with higher flooding tolerance, Glyma.03G00410, which presents two SNPs (Table 3), is located on chromosome 3 and encodes a calcium-binding protein, which converts signals in a wide variety of biochemical alterations [110]. Once bound to calcium, calmodulin acts as part of a signal transduction pathway, with various target proteins, such as kinases or phosphatases [65,111]. Thus, calmodulin can act as a mediator of soybean growth during stress [63].

In the adaptation of plants to flooding, the ethylene response factor (ERF) genes play an essential role in plant survival strategy through the regulation of gene expression [112]. The GSM0625 marker is in the Glyma.13G274100 gene, located on chromosome 13 (Table 3). This gene encodes transcription factors of the APETALA2/ethylene response factor (AP2/ERF) family. These transcription factors play key roles in signal transduction to activate or suppress the expression of defense genes, as well as act in growth regulation and the interaction between different signaling pathways and hormones in plants [113,114,115,116,117]. 

The AP2/ERF family has been characterized and its genes participate in the regulation of flooding tolerance [118]. After the onset of flooding, AP2/ERFs are induced and, in turn, interact with the *cis* elements present in the promoter of stress-responsive genes, providing greater tolerance to this condition [119]. Microarray studies have shown that several Arabidopsis AP2/ERF genes are induced during hypoxia [120]. Thus, AP2/ERF is regulated at different levels of transcription and translation to achieve homeostasis during adverse conditions, and is thus a promising candidate for the study of several networks involved in plant development, metabolic and stress responses.

The SNP GSM0605 is located in the Glyma.09G02100 gene, and this locus encodes a Cysteine desulfurase-like protein which is involved in the assembly of iron–sulfur (Fe–S) clusters [121]. These cofactors are needed in all clades of biology to perform a wide variety of unique functions, including nitrogen fixation, ribosome assembly, DNA repair, mitochondrial respiration, and metabolite catabolism. Fe–S proteins catalyze the conversion of the amino acid cysteine into alanine and elemental sulfur, and are known for their vital role in redox reactions during mitochondrial electron transport [122], biotin and thiamine formation, gene expression, and other cellular processes [123,124].

In addition to robust flooding tolerance assays to generate phenotypic data, genotypic assessments are also important for the development and selection of molecular markers. The outcomes of plant protein function and gene expression in response to a changing environment may result from SNPs in coding regions and regulatory sequences, respectively. The present study demonstrates that Brazilian soybean genotypes present allelic variation (Figure 2 and Table 9). 

The increase in the commercial value of soybeans on the international market has resulted in an increase in the cultivated area of this crop under different climatic and soil conditions in many regions of the world, including soils subject to periods of flooding [125]. A great challenge is the difficult adaptation of soybean to these conditions, since most genotypes are sensitive to excess water. A possible strategy is the identification and development of soybean genotypes more adapted to areas subject to flooding and that have a high grain yield potential. For this, it is important to recognize and understand the mechanisms of soybean response to flooding to improve grain productivity when grown in these areas. 

In Rio Grande do Sul state, Brazil, soybean cultivation is strongly associated with crop rotation with irrigated rice in lowland areas. In the 2021/2022 growing season, the area cultivated with soybeans in this environment in this state was approximately 380,000 ha. Thus, considering this important agricultural frontier for the expansion of soybean-cultivated areas, the obtention of genotypes with greater tolerance to flooding should be one of the goals of breeding programs [103,106,126,127,128]. This work provides some potential molecular markers that could be used for soybean genetic improvement and plant breeding. One of the advantages of SNP use is that the location of the variants in coding regions associated with biological and agronomic characteristics can be recognized, and phenotypes can be projected by genotypes, accelerating the selection of more tolerant plants to flooding.

## 3. Material and Methods

**Figure 4:**
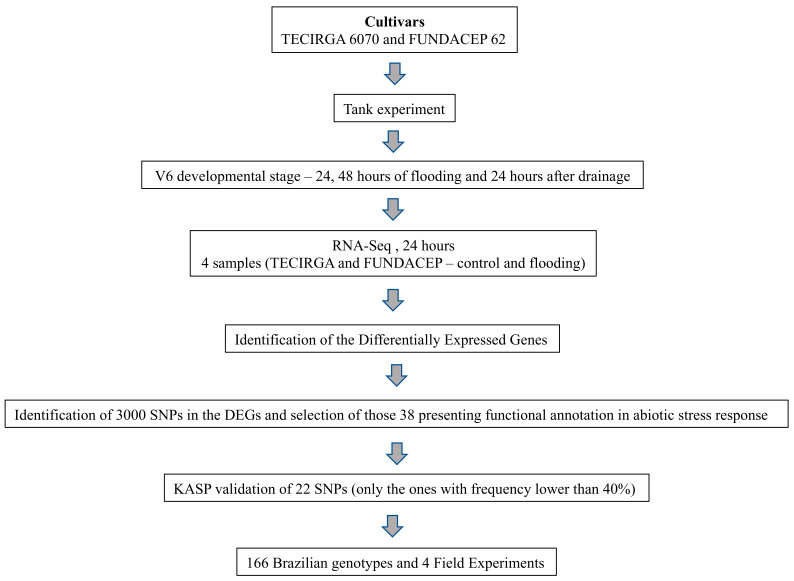
Experimental workflow.

A schematic representation of the experimental workflow is illustrated in Figure 4. The work started with an assay in tanks in which samples were collected for RNA sequencing. From the RNA-Seq data, SNPs were identified in differentially expressed genes related to the response to abiotic stress. Then, four field experiments were carried out to verify the tolerance of Brazilian genotypes to flooding.

### 3.1. Plant Material and Experiment Design

The first experiment was carried out from January to April 2014 under outdoor conditions at the Department of Crop Science, Federal University of Rio Grande do Sul, Porto Alegre, Brazil. Two indeterminate growth-type soybean genotypes with contrasting flooding tolerance (TECIRGA 6070RR-tolerant and FUNDACEP 62RR-sensitive) were selected for this study. TECIRGA was released as a flood-tolerant cultivar. Several experiments were carried out over three years, which made it possible to identify the tolerance to excess water of TECIRGA, which makes it suitable for cultivation in rice soils [129,130]. Plants from both genotypes were grown in 1.56 m^3^ concrete tanks (1.3 × 1.2 × 1.0 m) filled with a lowland gleysolic soil with the following physical and chemical characteristics: clay content = 170 g kg^−1^; pH = 5.4; phosphorus (P) = 70 mg dm^−3^; potassium (K) = 28 mg dm^−3^; organic matter = 20 g kg^−1^. In each tank, three rows (1.3 m long) of each genotype were sown on 13 January 2014, with a sowing density of 40 seeds m^−2^. Fertilization of 15, 90 and 45 kg ha^−1^ of nitrogen, phosphorus and potassium, respectively, was applied before sowing. The experiment was arranged in a randomized block design with three biological replicates. Weeds were controlled by hand and pest control practices were performed when necessary. 

Treatments consisted of two water regimes (flooded and control—not flooded) imposed 28 days after sowing when plants were at growth stage V6: six fully developed leaf nodes [131]. Both genotypes were submitted to flooding stress for 48 h using a water layer of approximately 5 cm. Control plants (not flooded), on the other hand, were not submitted to flooding and irrigated normally to maintain 85% field capacity. After flooding treatment, the tanks were drained. 

For physiological measurements and molecular analysis, leaves and roots were collected at three different time points: 24 and 48 h after the onset of flooding and 24 h after water drainage. Each sample represents a pool of two plants. Four samples were collected per time/cultivar/treatment (two samples for each tank). 

### 3.2. Total RNA Extraction and cDNA Synthesis

Samples were frozen in liquid nitrogen and stored at −80 °C for further RNA extraction. Total RNA was extracted from each sample using Trizol reagent (Invitrogen, Waltham, MA, USA). RNA quantification was performed using the NanoDrop 1000 spectrophotometer (Thermo Fisher Scientific, Waltham, MA, USA). The RNA integrity was analyzed using 260/280 nm ratio and confirmed by electrophoresis. Before cDNA synthesis, RNA was treated with DNase I (Invitrogen) according to the manufacturer’s instructions. First-strand cDNAs were obtained by using 1 μg of DNA-free RNA, M-MLV Reverse Transcriptase SystemTM (Invitrogen) and oligo (dT) primer.

### 3.3. RNA Sequencing and Sequence Analysis

Libraries were constructed for the two genotypes at two experimental conditions (control and 24 h of flooding) according to the manufacture’s recommendations. Samples were treated with DNase I and magnetic beads with Oligo (dT) were used to isolate mRNA. Mixed with the fragmentation buffer, the mRNA was fragmented into short fragments. Then, cDNA was synthesized using the mRNA fragments as templates. Short fragments were purified and resolved with EB buffer for end reparation and single nucleotide A (adenine) addition. Afterwards, the short fragments were connected with adapters. After agarose gel electrophoresis, the suitable fragments were selected for PCR amplification as templates. During the QC steps, Agilent 2100 Bioanaylzer and ABI StepOnePlus Real-Time PCR System were used in the quantification and qualification of the sample library. Four RNA samples were shipped as lyophilized material on RNA-stable tubes (Biomatrica) to the Beijing Genomics Institute—BGI (Beijing, China)—for RNA-Seq. All libraries were sequenced using the HiSeq™ 2000 at BGI.

Primary sequencing data produced were subjected to quality control (QC) and filtered into clean reads. The reads were aligned to the reference sequences with SOAPaligner/SOAP2 [132]. The results of gene expression included gene expression levels and differential expression analysis. Further Gene Ontology (GO) enrichment analysis and pathway enrichment analysis were performed.

### 3.4. PCR Primer Design

Primer pairs for qPCR were designed using the Primer Quest tool from IDT DNA (http://www.idtdna.com/primerquest/Home/Index, accessed on 14 April 2018) and checked for the presence of hetero and homodimers (Supplementary Table S1). To determine PCR efficiencies, standard curves were constructed with purified PCR products as a starting template, followed by 5 times 10-fold serial dilutions. Reaction efficiencies (E) and correlation coefficients (r^2^) were estimated using StepOne Software v. 2.3 (Life Technologies, Carlsbad, CA, USA), based on the slopes of the plots and the Cps (crossing points) versus log input of DNA. 

### 3.5. qPCR and Data Analyses

Reactions were performed with a StepOne Applied Biosystem Real-time Cycler^TM^ (Life Technologies). PCR-cycling conditions were implemented as described: 5 min 94 °C, followed by 40 cycles of 10 s at 94 °C, 15 s at 60 °C and 15 s at 72 °C. A melting curve analysis was performed at the end of the PCR run, over the range 55–99 °C, increasing the temperature stepwise by 0.1 °C every 1 s. Each 20 μL reaction comprised 10 μL cDNA (1:50 dilution), 1× PCR buffer (Invitrogen), 2.4 mM MgCl_2_, 0.4 mM dNTP, 0.1 M each primer, 0.1× SYBR-Green I (Invitrogen) and 0.3 U of Platinum Taq DNA Polymerase (Invitrogen). All PCR reactions were performed in technical quadruplicates. The expression data analyses were performed after the comparative quantification of amplified products using the 2^−ΔΔCt^ method [133].

In order to select the appropriate reference genes, all results from qPCR were compared using two different algorithms: NormFinder [134] and geNorm [135]. 

### 3.6. Agronomic and Physiological Evaluations

In order to characterize flooding stress and plant responses, in vivo chlorophyll fluorescence, ascorbate peroxidase (APX) activity, hydrogen peroxide content, nitrogen uptake, canopy reflectance (NDVI), grain production and yield components were evaluated. Specifications about the variables analyzed in each experiment are described in Table 11.

Ascorbate peroxidase (APX) activity and hydrogen peroxide content: APX activity was assayed from the total soluble protein extract. The protein extraction was performed utilizing fresh leaf matter in the presence of 100 mM phosphate buffer pH 7. Protein content was measured by Bradford assay using bovine serum albumin as standard. The activity of APX (EC 1.11.1.1) was assayed in the total soluble extract. The reaction mixture contained 50 mM phosphate buffer pH 7, 500 µM AsA, 1 mM H_2_O_2_ and 100 µg of protein total extract. APX activity was determined following the depletion in absorbance at 290 nm using a spectrophotometer. The extinction coefficient of AsA (2.62 mm^−1^ mm^−1^) was used to calculate the APX activity. 

The hydrogen peroxide (H_2_O_2_) was detected with Ampiflu™ Red (Sigma Aldrich) according to the manufacturer’s instructions. The reagent was used in combination with horseradish peroxidase (HRP) to detect H_2_O_2_ released from the total soluble protein extract.

Chlorophyll fluorescence: The effective photosystem II quantum yield, which estimates the maximum quantum efficiency of PSII, was determined under natural light conditions with a portable pulse modulation fluorometer (Model OS1-FL, Opti-Sciences, Hudson, NY, USA) by the following equation: YieldPSII = Fm′ − Ft/Fm′, where Fm′ is the maximal fluorescence which is induced with a saturating white light pulse (400 ms, approximately 4000 μmol m^−2^ s^−1^) and Ft is the current steady-state fluorescence in light-adapted leaves. Measurements were performed before the onset of flooding (10 February) and afterwards daily for four days. Readings were carried out in the same plants and at the same time (11:00 AM) on the adaxial surface of the uppermost, fully expanded leaf in five plants per replicate.

Quantification of canopy reflectance: Canopy reflectance was measured using the Greenseeker^®^ hand-held sensor which provides the value of the normalized difference vegetation index (NDVI) [136] by the relation: (ρNIR − ρR)/(ρNIR + ρR), where ρNIR and ρR denote the near-infrared (780 nm) and red (670 nm) reflectance, respectively. The equipment was positioned at 1.0 m above the canopy, and readings were obtained for the entire row, totaling approximately 120 NDVI values in each experimental unit.

Nitrogen uptake: Nitrogen (N) uptake was evaluated by sampling and drying two plants per replicate at 60 °C up to constant weight. Afterwards, shoot N content was determined by the Kjeldahl method [137]. Plant N uptake was calculated by multiplying shoot dry biomass by its nitrogen content. Plants were sampled before the onset of flooding, two days after the tank´s drainage and at flowering (growth stage R1). 

Grain yield was evaluated (Experiments 2 and 3) by harvesting an area of 3 m^2^. The weight of seeds per experimental unit was quantified and the value was extrapolated to seed yield in kg ha^−1^ (on a 13% moisture basis).

### 3.7. Plant Mortality and Leaf Retention

Plant mortality was determined by means of visual evaluation, in the beginning of the legume formation (growth stage R3) and at harvest (growth stage R8), using a scale ranging from zero (no living plants in the plot) to 100% (all living plants on the plot). The visual evaluation of foliar retention was performed at harvest (growth stage R8) using a scale ranging from zero (no green leaves in the plant) to five (all green leaves in the plant).

### 3.8. Statistical Analysis

Chlorophyll fluorescence, N uptake, grain production and yield component data were subjected to analysis of variance (ANOVA). When F-test was significant (*p* < 0.05), comparison of means was performed by Student’s *t*-test using the software SPSS Statistics for Windows, version 12.0 (SPSS Inc., Chicago, IL, USA).

### 3.9. Single-Nucleotide Polymorphism Analysis

Based on the dataset generated through RNA sequencing, SNPs were then identified. The SNPs in differentially expressed genes in response to flooding were identified using the SAMtools method [138]. The reads of each library were anchored to the soybean genome (*Glycine max* Wm82.a2.v1) with the BWA software [139] to generate BAM files. From these files, SNPs with a minimum coverage of 20 reads were identified in each library. Only SNPs polymorphic between the tolerant genotype (TECIRGA 6070) and sensitive genotype (FUNDACEP 62) were selected. Of these pre-selected SNPs, only SNPs in genes with functional annotation to respond to abiotic stresses were filtered out using the Blast2GO platform (https://www.blast2go.com/, accessed on 17 November 2018) to reduce the number of candidate genes. To validate the polymorphism to be used as a molecular marker, SNPs were selected with allele frequencies lower than 40% at the Phytozome database (available at https://phytozome.jgi. doe.gov/pz/portal.html, accessed on 3 August 2019). 

### 3.10. KASP Assay Design and SNP Validation

To test the association of SNPs with the flooding tolerance and develop robust markers for marker-assisted selection, competitive allele-specific PCR (KASP) assays were developed from these SNPs and tested using two different approaches: (i) a bi-parental population, F_5_-derived recombinant inbred lines (RILs) from Benning × PI 416,937 and (ii) a panel of 166 Brazilian soybean genotypes. The assays were performed as previously described [140]. 

From a panel of 11 contrasting genotypes with a known phenotype for flood tolerance, the 22 SNP markers designed for allele competition assay were tested for the initial evaluation of promising markers. The genotypes used were Williams 82 (reference genome) NA 5959, NA 5909, SYN 1359, Woodruff, Boog and PI 416937, considered sensitive; Valente and Ponta, considered intermediate; and TECIRGA 6070 and Benning, considered tolerant to excess water. After this genotyping, 8 SNP markers that showed common polymorphism in the tolerant genotypes, TECIRGA 6070 and Benning, were selected.

The phenotypic data from F5-derived recombinant inbred lines from Benning (tolerant) × PI 46,937 (sensitive) were collected in 2012, 2014 and 2015 at Stuttgart, AR (USA). Flooding treatment was applied at R1 stage for 11 days. The plant damage by flooding stress was rated three times visually on a scale from 0 to 9 (where 0 = no damage and 9 = 100% plants dead), and as percent of plant survival. The 128 individuals derived from the Benning × PI 46,937 cross was performed for the 8 SNPs previously selected. 

### 3.11. Field Experiments—Brazilian Soybean Genotypes

Four experiments were carried out at the Experimental Rice Station of the Instituto Rio Grandense do Arroz (EEA/IRGA), in Cachoeirinha, State of Rio Grande do Sul, Brazil, during the 2017/18 and 2018/19 growing seasons on gleysol soil (lowland soil). Plants were submitted to flooding by using a water layer of approximately 5 cm above soil level. After flooding stress submission, water outlets of the plots were opened for drainage. 

Experiments 1, 2 and 3 were conducted in a randomized block experimental design with four replicates. Each plot was composed of four rows of 6 m in length, with a row spacing of 0.5 m, constituting 12 m^2^. At Experiment 4, each experimental unit consisted of an area of 3 m^2^, consisting of two lines 3 m long, spaced 0.5 m apart. The experimental design used was also the randomized blocks, with two repetitions. The fertilization of the experimental area was carried out with application in lines: 126 kg ha^−1^ of P_2_O_5_ (triple superphosphate) and 120 kg ha^−1^ of K_2_O (potassium chloride) after the sowing. Soybean seeds were previously treated with fungicide and insecticide based on pyraclostrobin (25 g l^−1^), thiophanate-methyl (225 g l^−1^) and fipronil (250 g l^−1^) at the dose of 2 mL of commercial product kg^−1^ of seed. Experiment 1, which was conducted in the 2017/18 season, included 105 genotypes and the flooding stress was imposed when the plants were at the V7-V8 growth stage. After six days of flooding treatment, phenotyping (visual scale) was performed. Experiment 2 was also conducted in 2017/18, and included 32 genotypes; the duration of stress was five days, and three phenotyping studies were carried out, which varied from 8 to 43 days after the end of the flooding period. With the size of the plots in this experiment, soybean seed yield was also taken. The third experiment included 26 genotypes and was carried out in the 2018/19 growing season. Plants were grown up to the V7-V8 growth stage and flooded for a period of three days. Four phenotyping ratings were taken, which varied from 15 to 60 days after the end of the flooding period. In addition to the phenotyping ratings, mortality, NDVI, leaf retention and grain yield were also collected. The fourth experiment was also carried out in the 2018/19 growing season and included 77 genotypes. Flooding was imposed when the plants were at the V6-V8 growth stage and lasted for five days. Three phenotyping studies were performed during the first 10 days and the last 38 days after water drainage. 

In total, 166 soybean genotypes from 14 different companies were tested, and 41 of those were used in more than one experiment (Supplementary Table S2). For phenotyping the plant response to flooding, a visual scale was taken with ratings ranging from 0 to 9, where zero corresponds to plants without symptoms of stress and nine corresponds to all plants being dead. The phenological scale used was that proposed by Fehr and Caviness [131].

### 3.12. Statistical Analysis

A phenotypic rating for flooding tolerance among the experiments was used for analysis of variance (ANOVA) using PROC GLM in SAS v9.4 (SAS Institute Inc., Cary, NC, USA). The BLUPs were calculated by treating replication and experiments as fixed effects, and the genotype and genotype by experiment interactions as random effects to help account for variation among replications and experiments. The BLUPs of these genotypes were then used to test the association with the SNP genotypes in SAS version 9.4. Genome-wide association analysis was performed using a ‘fixed and random model circulating probability unification’ (FarmCPU) method [141]. 

## Figures and Tables

**Table 1:** Output statistics of transcriptome sequencing.

	Genotypes	Genome Map Rate (%)	Gene Map Rate (%)	Expressed Genes	Novel Transcripts	Alternative Splicing
**Flooding**	TECIRGA 6070	82.26	90.84	53,585	441	57,741
	FUNDACEP 62RR	82.27	91.16	53,226	416	54,951
**Control**	TECIRGA 6070	81.76	91.15	53,577	411	58,044
	FUNDACEP 62RR	82.07	91.52	53,677	409	55,495

**Table 2:** Differential expression statistics.

	Flooding	Control	Flooding vs. Control	
	TECIRGA 6070 vs. FUNDACEP 62RR *		TECIRGA 6070	FUNDACEP 62RR
Number of genes up-regulated	752	1494	1356	2242
Number of genes down-regulated	871	731	1278	1052

* Comparison between genotypes in each condition.

**Table 3:** List of SNPs selected for KASP genotyping.

Molecular Marker	Locus	SNP Position	Annotation
**GSM0599**	Glyma.02G04840 0	Ch02:4442101	Flavanone 3-dioxygenase
**GSM0600**	Glyma.18G15870 0	Ch18:35426853	Alcohol dehydrogenase
**GSM0601**	Glyma.18G22310 0	Ch18:50992583	Geraniol 8-hydroxylase/G10H
**GSM0602**	Glyma.02G26780 0	Ch02:45217239	E3 ubiquitin-protein ligase RFWD2 (RFWD2, COP1)
**GSM0603**	Glyma.11G22880 0	Ch11:32402605	Chlorophyll a-b-binding chloroplastic
**GSM0604**	Glyma.18G24800 0	Ch18:53508659	Alcohol dehydrogenase transcription factor MYB/SANT-like protein
**GSM0605**	Glyma.09G02100 0	Ch09:1660885	Cysteine desulfurase/selenocysteine lyase (sufS)
**GSM0611**	Glyma.04G00040 0	Ch04:54135	Alpha-1,3-mannosyl-glycoprotein 2- beta-N-acetylglucosaminyltransferase/1,2-N-acetylglucosaminyltransferase
**GSM0612**	Glyma.03G00410 0	Ch03:366065	Calmodulin-1 related
**GSM0613**	Glyma.03G00410 0	Ch03:366478	
**GSM0614**	Glyma.05G21320 0	Ch05:39469081	Calcium-dependent kinase 3-like
**GSM0615**	Glyma.06G01940 0	Ch06:1475861	ATP-dependent zinc metalloprotease chloroplastic
**GSM0616**	Glyma.10G10610 0	Ch10:23946187	Translation initiation factor 3 subunit E (EIF3E, INT6)
**GSM0617**	Glyma.12G19010 0	Ch12:35169651	DnaJ homolog
**GSM0618**	Glyma.12G19010 0	Ch12:35171053	
**GSM0619**	Glyma.13G18950 0	Ch13:30296886	cysteine ase inhibitor 6
**GSM0620**	Glyma.13G28200 0	Ch13:38340820	Chlorophyll a-b-binding chloroplastic
**GSM0621**	Glyma.13G28200 0	Ch13:38342317	
**GSM0622**	Glyma.14G20300 0	Ch14:46789605	CBL-interacting serine threonine- kinase 11-like
**GSM0623**	Glyma.18G09150 0	Ch18:9144124	Cinnamyl-alcohol dehydrogenase
**GSM0625**	Glyma.13G27410 0	Ch13:37559221	AP2 domain (AP2)
**GSM0626**	Glyma.13G23390 0	Ch13:34460517	Ethylene-responsive binding factor 1

**Table 4:** Alleles significantly associated with visual scores and plant survival rate in the years 2014 and 2015.

Molecular Marker		Visual Score		Plant Survival			
	SNP Position *	2012	2014	2015	2012	2014	2015
	R^2^ (*p*-Value)						
GSM0612	Gm03: 366065	n.s.	8% (*p* < 0.003)	8% (*p* < 0.002)	n.s.	12% (*p* < 0.003)	6% (*p* < 0.018)
GSM613	Gm03: 366478	n.s.	10% (*p* < 0.001)	8% (*p* < 0.003)	n.s.	15% (*p* < 0.001)	6% (*p* < 0.012)

* Represents the SNP coordinate in the *Glycine max* genome (Wm82.a2.V1); n.s. = not significant.

**Table 5:** Associative analysis of SNP markers and phenotyping (visual score and productivity) of soybean Brazilian genotypes submitted to flooding (Experiments 1 and 2).

	Alleles	Experiment 1			Experiment 2								
			Phenotyping 1			Phenotyping 1		Phenotyping 2		Phenotyping 3		Productivity	
		N ^(1)^	M ^(2)^	*p*-Value	N ^(1)^	M ^(2)^	*p*-Value	M ^(2)^	*p*-Value	M ^(2)^	*p*-Value	M ^(2)^	*p*-Value
GSM600	AA	62	7.86	0.01									
	CC	33	8.15										
GSM601	TT	79	7.9	0.01	23	6.03	0.01	6.53	0.01	6.53	0.00	1357.26	0.01
	AA	17	8.24		9	5.90		5.39		5.57		1807.69	
GSM602	TT	60	8.03	0.05								1591.09	0.02
	AA	36	7.84									1222.54	
GSM605	TT	24	7.66	0.00									
	GG	70	8.06										
GSM616	TT	62	8.08	0.00	23	6.59	0.04						
	CC	32	7.76		9	6.00							
GSM620	GG	81	7.97	0.05									
	AA	2	7.31										
GSM621	AA	94	7.97	0.05									
	TT	2	7.31										
GSM623	TT	24	8.14	0.04									
	AA	72	7.9										
GSM625	GG	74	8.07	0.00	20							1378.23	0.05
	AA	22	7.58		12							1660.12	
GSM604	TT				23	6.70	0.00	5.43	0.00	6.52	0.00	1385.58	0.02
	AA				8	5.64		5.20		5.41		1777.87	
GSM612	TT				12	6.06	0.03	5.53	0.01	5.72	0.00	1770.12	0.00
	CC				20	6.65		6.42		6.58		1312.23	
GSM613	GG				12	6.06	0.04	5.53	0.01	5.72	0.00	1770.12	0.00
	AA				19	6.57		6.37		6.58		1347.16	
GSM619	TT				30			6.18	0.02				
	GG				2			4.63					

^(1)^ Number of genotypes classified in each allele group and ^(2)^ average grade of the visual damage scale.

**Table 6:** Associative analysis of SNP markers for phenotyping (visual score) of Brazilian soybean genotypes under flooding conditions (Experiments 3 and 4).

			Experiment 3								Experiment 4						
Marker	Alleles		Phenotyping 1		Phenotyping 2		Phenotyping 3		Phenotyping 4		Phenotyping 1			Phenotyping 2		Phenotyping 3	
		N ^(1)^	M ^(2)^	*p*-Value	M ^(3)^	*p*-Value	M ^(4)^	*p*-Value	M ^(5)^	*p*-Value	N ^(1)^	M ^(6)^	*p*-Value	M ^(7)^	*p*-Value	M ^(8)^	*p*-Value
GSM599	CC	24			5.84	0.03											
	GG	2			5.06												
GSM604	TT										51					6.88	0.03
	AA										22					7.27	
GSM605	TT										23	6.41	0.00	6.53	0.00	6.59	0.00
	GG										50	7.09		7.08		7.14	
GSM611	TT	18							5.56								
	CC	6							6.29	0.02							
GSM612	TT	9			5.54	0.03	5.63	0.02	5.40	0.04							
	CC	15			5.98		6.16		5.99								
GSM613	GG	9			5.54	0.04	5.38	0.00	5.40	0.05							
	AA	15			5.95		6.13		5.95								
GSM616	TT	19							5.56	0.03							
	CC	6							6.23								
GSM622	GG	12	5.82	0.02													
	AA	14	6.24														
GSM625	GG	11							6.07	0.03							
	AA	15							5.49								

^(1)^ Number of genotypes classified in each group and ^(2–5)^ average of visual damage scales performed 3, 15, 29, 43 and 60 days after drainage, respectively, from Experiment 3 in the season 2018/19; ^(6–8)^ average of visual damage scales performed 10, 23 and 38 days after drainage, respectively, from Experiment 4 in the 2018/19 season.

**Table 7:** Association of SNPs and productivity (kg ha^−1^), mortality (%), NDVI and leaf retention variables in different Brazilian soybean genotypes under flooded conditions.

			Experiment 3								Experiment 4				
Marker	Alleles		Productivity		Mortality		NDVI		Foliar Retention		Mortality			NDVI	
		N ^(1)^	M ^(2)^ (kg ha^−1^)	*p*-Value	M ^(3)^ (%)	*p*-Value	M ^(4)^	*p*-Value	M ^(5)^	*p*-Value	N ^(1)^	M ^(6)^ (%)	*p*-Value	M ^(7)^	*p*-Value
GSM0604	TT										51			0.46	0.04
	AA										22			0.43	
GSM0605	TT	10					0.62	0.04	0.59	0.01	23	47.65	0.03	0.47	0.02
	GG	13					0.66		1.32		50	55.94		0.44	
GSM0611	TT	18	1175.4	0.04					1.13	0.03					
	CC	6	875.0						0.50						
GSM0612	TT										32	49.38	0.02		
	CC										41	58.22			
GSM0613	GG										32	49.38	0,03		
	AA										39	58.05			
GSM0614	CC										26	48.35	0.02		
	AA										49	57.12			
GSM0618	TT										21			0.47	0.04
	CC										52			0.44	
GSM0625	GG	11	918.63	0.02											
	AA	15	1214.7												

^(1)^ Number of genotypes classified in each allele group; ^(2–5)^ average productivity, mortality, NDVI and leaf retention, respectively, of soybeans in the 2018/19 season, Experiment 3. ^(6,7)^ Average mortality and NDVI, respectively, of soybeans in the 2018/19 season, Experiment 4. Values in bold highlight *p*-values < 0.05 obtained by *t*-test.

**Table 8:** Significant correlation between SNP markers and the variables analyzed in each experiment.

Molecular Marker	Experiment 1	Experiment 2				Experiment 3								Experiment 4				
	Phenotyping	Phenotyping			Productivity	Phenotyping				Productivity	Mortality	NDVI	Foliar Retention	Phenotyping			Mortality	NDVI
	1	1	2	3		1	2	3	4					1	2	3		
GSM0599							X											
GSM0600	X^1^																	
GSM0601	X	X	X	X	X													
GSM0602	X				X													
GSM0603																		
GSM0604		X	X	X	X											X		X
**GSM0605**	X	X	X									X	X	X	X	X	X	X
**GSM0611**									X	X			X					
**GSM0612**		X	X	X	X	X	X	X									X	
**GSM0613**		X		X	X	X	X	X									X	
GSM0614																	X	
GSM0615																		
GSM0616	X	X						X										
GSM0617																		
GSM0618																		X
GSM0619			X															
GSM0620	X																	
GSM0621	X																	
GSM0622						X												
GSM0623	X																	
**GSM0625**	X				X				X	X								
GSM0626	X																	

**Table 9:** Genotypes associated with greater tolerance to flooding for the five SNPs previously selected and evaluated.

Genotype	GSM0605 (TT)	GSM0611 (TT)	GSM0612 (TT)	GSM0613 (GG)	GSM0625 (AA)	Associated Markers
BRB 16-253244	TT	TT	TT	GG	AA	5
HO TERERÊ	TT	TT	TT	GG	AA	5
SYN 1451946	TT	TT	TT	GG	AA	5
BRB 16-238251	TT	TT	TT	GG	AA	5
NS 6700	TT	TT	TT	GG	AA	5
BRB 16-236559	TT	TT	TT	GG	GG	4
TMG 7058	GG	TT	TT	GG	AA	4
BRB 16-242992	GG	TT	TT	GG	AA	4
PIO 95R95	TT	TT	TT	GG	GG	4
BS 1691	GG	TT	TT	GG	AA	4
DM 66I68	GG	TT	TT	GG	AA	4
SYN 1561	GG	TT	TT	GG	AA	4
TEC 7849	GG	TT	TT	GG	AA	4
M 5838	TT	TT	TT	GG	GG	4
M 6410	TT	CC	TT	GG	AA	4
BMX PONTA	TT	CC	TT	GG	AA	4
CZ 26B42	GG	TT	TT	GG	AA	4
M 5947	TT	TT	TT	GG	GG	4
BRB 16-244779	TT	TT	CC	AA	AA	3
BRB 16-248756	TT	TT	CC	AA	AA	3
BRB 12248755	TT	TT	CC	AA	AA	3
BRB 16-237636	GG	CC	TT	GG	AA	3
NA 5909	GG	CC	TT	GG	AA	3
TMG 1759	GG	CC	TT	GG	AA	3
BRB 16-252363	TT	CC	TT	GG	GG	3
BRB 16-237091	GG	CC	TT	GG	AA	3
BRB 16-237639	GG	CC	TT	GG	AA	3
BRB 16-237622	GG	CC	TT	GG	AA	3
DS 6017	GG	CC	TT	GG	AA	3
BRB 16260221	TT	TT	CC	AA	AA	3
DM 5958	TT	CC	TT	GG	GG	3
BRB 16-262006	GG	TT	TT	GG	GG	3
BRS 16-258758	GG	TT	TT	GG	GG	3
SYN 63S38	GG	TT	TT	GG	GG	3
TMG 1264	GG	TT	TT	GG	GG	3
SYN 1263	GG	CC	TT	GG	AA	3
BRB-16259264	TT	TT	CC	AA	AA	3
TMG 7063	TT	TT	CC	AA	AA	3
TMG 7061	TT	TT	CC	AA	AA	3
BS IRGA 1642	TT	TT	CC	AA	AA	3
BRB 16-258183	TT	CC	TT	GG	GG	3
BMX VALENTE	GG	TT	TT	GG	GG	3
NS 6209	GG	TT	TT	GG	GG	3
BRB 16239550	TT	CC	TT	GG	GG	3
TEC IRGA 6070	TT	CC	TT	GG	GG	3
NS 6601	GG	TT	TT	GG	GG	3
BRB 16-238969	GG	TT	TT	GG	GG	3
CD 2737	GG	TT	TT	GG	GG	3
GMX CANCHEIRO	GG	TT	TT	GG	GG	3
BMX ÍCONE	TT	TT	CC	AA	AA	3
PF 121217	GG	TT	TT	GG	GG	3
BRR 16-109850	GG	TT	TT	GG	GG	3
BRS 5804	GG	TT	TT	GG	GG	3

**Table 10:** Single-factor analysis results using both BLUPs and fixed effects.

Chrom.	Marker	b0	b1	−2ln(L0/L1)	F(1,n−2)	pr(F)	R2
2	GSM0599	6.728	−0.075	0.319	0.315	0.575306313	0
2	GSM0602	6.768	0.064	1.67	1.658	0.199725006	0.0009
3	GSM0612	6.788	−0.06	1.142	1.132	0.288859754	0.0028
3	GSM0613	6.79	−0.061	1.209	1.199	0.275156569	0.0022
4	GSM0611	6.787	−0.09	2.614	2.603	0.108562391	0.0114
5	GSM0614	6.798	−0.002	0.001	0.001	0.976409918	0.0005
6	GSM0615	6.801	−0.023	0.168	0.167	0.683769788	0.0019
9	**GSM0605**	6.731	−0.169	8.148	8.251	0.004612844 **	0.0594
10	**GSM0616**	6.762	0.136	5.763	5.793	0.017197304 *	0.0276
11	GSM0603	6.797	−0.016	0.079	0.078	0.77987755	0.0006
12	GSM0617	6.822	0.06	0.971	0.963	0.327989769	0.0052
12	GSM0618	6.813	0.032	0.25	0.247	0.619632121	0.0017
13	GSM0619	6.786	0.014	0.033	0.032	0.857865418	0.0004
13	GSM0626	6.795	0.008	0.018	0.018	0.893648882	0.0004
13	**GSM0625**	6.754	0.117	4.053	4.053	0.045728869 *	0.0214
13	GSM0620	6.791	0.008	0.008	0.008	0.930929435	0.0029
13	GSM0621	6.395	−0.41	1.732	1.721	0.191460509	0.0033
14	GSM0622	6.798	−0.006	0.014	0.014	0.906612065	0
18	GSM0623	6.82	0.071	2.113	2.101	0.149127453	0.02
18	GSM0600	6.84	0.079	1.567	1.556	0.214085886	0.0093
18	GSM0601	6.823	−0.042	0.428	0.424	0.516020258	0.0011

* *p* < 0.05, ** *p* < 0.01.

**Table 11:** Phenotyping and physiological analyses performed.

		Experiment			
	Tank (Outdoor)	Field			
	RNA-Seq	1	2	3	4
Genotypes	2	105	32	26	77
Season	2014	2017/18	2017/18	2018/19	2018/19
Developmental stage of flooding	V6	V7-V8	V7-V8	V7-V8	V6-V8
Flooding period	24 and 48 h	5 days	5 days	3 days	5 days
APX activity and H_2_O_2_	A	-	-	-	-
Chlorophyll fluorescence	A	-	-	-	-
Nitrogen uptake	A	-	-	-	-
Phenotyping 1 (days after drainage)	-	6	8	15	10
Phenotyping 2 (days after drainage)	-	-	23	29	23
Phenotyping 3 (days after drainage)	-	-	43	43	38
Phenotyping 4 (days after drainage)	-	-	-	60	-
NDVI	-	-	-	A	A
Plant mortality	-	-	-	A	A
Foliar retention	-	-	-	A	-
Grain yield	-	-	A	A	-

-: not analyzed; A: analyzed.

## Supplementary Materials

The supporting information can be downloaded at: https://www.mdpi.com/article/10.3390/ijms231810611/s1.
