# Supplementary material for: Identification of Functional Genetic Variations Underlying Flooding Tolerance in Brazilian Soybean Genotypes
Source: Int J Mol Sci. 2022 Sep 13;23(18):10611. doi: 10.3390/ijms231810611 (PMC9502317; doi:10.3390/ijms231810611)
Supplement: Supplementary file 1 [file ijms-23-10611-s001.zip › Supplemenatry Table S9.pdf]

Supplementary Table S9. Association of SNPs and mortality and NDVI variables in different Brazilian soybean genotypes under flooding conditions.

Experiment 4					
**Molecular Marker**	N(1)	**Mortality**		**NDVI**	
		M(2) (%)	*p-value*	M(3)	*p-value*
**GSM0599**					
CC	74	54.41	0.44	0.45	0.40
GG	2	52.5		0.44	
**GSM0600**					
AA	65	54.6	0.4	0.45	0.35
CC	11	53.1		0.44	
**GSM0601**					
TT	53	53.3	0.23	0.45	0.29
AA	22	57		0.44.	
**GSM0602**					
TT	52	53.4	0.45	0.45	0.18
AA	17	54.1		0.44	
**GSM0603**					
TT	37	52.8	0.29	0.46	0.19
GG	36	55.2		0.44	
**GSM0604**					
TT	51	52.9	0.16	0.46	**0.04**
AA	22	57.7		0.43	
**GSM0605**					
TT	23	47.65	**0.03**	0.47	**0.02**
GG	50	55.94		0.44	
**GSM0611**					
TT	45	53.47	0.48	0.46	0.07
CC	28	53.74		0.44	
**GSM0612**					

TT	32	49.38	**0.02**	0.45	0.3
CC	41	58.22		0.44	
**GSM0613**					
GG	32	49.38	**0.03**	0.45	0.33
AA	39	58.05		0.45	
**GSM0614**					
CC	26	48.35	**0.02**	0.46	0.27
AA	49	57.12		0.45	
**GSM0615**					
GG	42	53.07	0.38	0.46	0.18
AA	29	54.31		0.44	
**GSM0616**					
TT	42	55.26	0.32	0.45	0.42
CC	31	53.23		0.45	
**GSM0617**					
TT	23	53.91	0.49	0.47	0.05
AA	50	54		0.44	
**GSM0618**					
TT	21	53.33	0.33	0.47	**0.04**
CC	52	55.44		0.44	
**GSM0619**					
TT	73	54.05	0.24	0.45	0.06
GG	3	61.67		0.4	
**GSM0620**					
GG	74	45	nd	0.46	0.36
AA	1	54.74		0.45	
**GSM0621**					
AA	75	45	nd	0.46	0.36
TT	1	54.48		0.45	
**GSM0622**					
GG	43	53	0.29	0.44	0.39

AA	29	55.45		0.45	
**GSM0623**					
TT	22	53.1	0.23	0.45	0.49
AA	50	56.59		0.45	
**GSM0625**					
GG	46	52.8	0.22	0.46	0.12
AA	28	56.29		0.44	
**GSM0626**					
GG	47	53.4	0.28	0.45	0.39
AA	29	55.9		0.45	

(1) Number of genotypes classified in each group and (2) (3) Average mortality and NDVI, respectively, of soybean in the 2018/19 season, experiment 4. nd: data not available. Values in bold highlights p-values < 0.05 obtained by t-test.
