# Supplementary material for: Identification of Functional Genetic Variations Underlying Flooding Tolerance in Brazilian Soybean Genotypes
Source: Int J Mol Sci. 2022 Sep 13;23(18):10611. doi: 10.3390/ijms231810611 (PMC9502317; doi:10.3390/ijms231810611)
Supplement: Supplementary file 1 [file ijms-23-10611-s001.zip › Supplementary Table S1.pdf]

Supplementary Table S1: List of primer sequence and related information for each gene.

Name	Locus ID	Annotation	Sequence 5'-3'	Amplicon size (bp)	Reference
LCT 06	Glyma.05G063600	Ethylene-responsive transcription factor 15-related	F: GGCGAGTCCGAATCCATATT<br>R: CCCAATTGTCGCTCAAACAC	94	This study
LCT 07	Glyma.08G189600.1	Linoleate 9S-lipoxygenase / Linoleate 9-lipoxygenase	F: ATATCCTTAACCGGCCAACTC<br>R: GGCATCACTCTTCCCTGTAAT	125	This study
LCT 09	Glyma.09G041500.1	AP2 domain (AP2)	F: GACGAACTCGACCTGAAACA<br>R: CATCCATGTTGTCCCACTCA	97	This study
LCT 10	Glyma.02G257700.1	Leucocyanidin oxygenase / Leucoanthocyanidin dioxygenase	F: ATACCCATAGAGCCAGCAAAG<br>R: CGCATGGTCCTCTCAATCTAAT	109	This study
LCT 11	Glyma.14G042200.1	Alcohol dehydrogenase related	F: GCCAGGTTCTCCATTGAGTATT<br>R: TCGCTGTGACAAAGAGAAGTG	99	This study
LCT 12	Glyma.14G121200.1	Alcohol dehydrogenase related	F: GGTGATTGAAGAAGTGGAGGT<br>R: GTGGCAAAGAGAGGTGTAGAG	79	This study
ETR	Glyma.19G213300	Two-component sensor histidine kinase	F: ATGGATGCCTTCAAGAAGTGG<br>R: GCACATATCTTCCCACAAGAGG	188	Arraes *et al.* 2015
ACO	Glyma.14G048900	1-aminocyclopropane-1-carboxylate oxidase 3-related	F: CCAATGCGCCATTCCATTGTTG<br>R: TGAGGCTACGGACATTCTGGTC	126	Arraes *et al.* 2015
Fbox	Glyma.12G051100	F-Box only protein 3	F: GATAGGGAAATGTTGCAGGT<br>R: CTAATGGCAATTGCAGCTCTC	93	Le *et al*. 2012
CYP2A	Glyma.12G024700	Peptidyl-prolyl cis-trans isomerase CYP18-3-related	F: CGGGACCAGTGTGCTTCTTCA<br>R:CCCCTCCACTACAAAGGCTCG	154	Miranda *et al*. 2013

---Internal Use---

ELF 1A	Glyma.05G114900	Elongation factor 1-alpha	F: GACCTTCTTCGTTTCTCGCA<br>R: CGAACCTCTCAATCACACGC	195	Miranda *et al*. 2013
ELF 1B	Glyma.02G276600	Elongation factor 1-beta 1-related	F: GTTGAAAAGCCAGGGGACA<br>R: TCTTACCCCTTGAGCGTGG	118	Miranda *et al*. 2013
TUA5	*	Tubulin	F: AGGTCGGAAACTCCTGCTGG<br>R: AAGGTGTTGAAGGCGTCGTG	159	Miranda *et al*. 2013
UBC4	*	Ubiquitin conjugating enzyme	F: GAGCGAGCAGTTTCAGAC<br>R: CATAGGAGGGACGATACG	168	Miranda *et al*. 2013
UBC2	Glyma.18G124600	Ubiquitin conjugating enzyme	F: TCCCCTCACACCCTTCCTC<br>R: CCATCCCAAGGGGTGTCAT	155	Miranda *et al*. 2013
ACT11	Glyma.18G290800	Actin	F: CGGTGGTTCTATCTTGGCATC<br>R: GTCTTTCGCTTCAATAACCCTA	142	Miranda *et al*. 2013
G6PD	*	Glucose-6-phosphate dehydrogenase	F: ACTCCTTGATACCGTTGTCCAT<br>R: GTTTGTTATCCGCCTACAGCCT	126	Miranda *et al*. 2013
Isoflavone	Glyma.06G030900	Isoflavone	F: CGAGTTTCTGCCTCTGCTTT<br>R: CCCATTGAAGTTTCTGTTACTGC	91	Khatoon *et al*. 2012
