# Supplementary material for: Identification of Functional Genetic Variations Underlying Flooding Tolerance in Brazilian Soybean Genotypes
Source: Int J Mol Sci. 2022 Sep 13;23(18):10611. doi: 10.3390/ijms231810611 (PMC9502317; doi:10.3390/ijms231810611)
Supplement: Supplementary file 1 [file ijms-23-10611-s001.zip › Supplementary Table S4.pdf]

Table S4. Associative analysis of SNPs markers and phenotyping (visual score) of soybean Brazilian genotypes submitted to flooding (Experiment 1).

Experiment 1							
**Molecular Marker**	**Phenotyping**			**Molecular Marker**	**Phenotyping**		
	N(1)	M(2)	*p-value*		N(1)	M(2)	*p-value*
**GSM0599**				**GSM0615**			
CC	92	7.97	0.46	GG	54	7.94	0.32
GG	3	7.93		AA	41	7.99	
**GSM0600**				**GSM0616**			
AA	62	7.86	**0.01**	TT	62	8.08	**0.00**
CC	33	8.15		CC	32	7.76	
**GSM0601**				**GSM0617**			
TT	79	7.9	**0.01**	TT	30	8.08	0.09
AA	17	8.24		AA	63	7.91	
**GSM0602**				**GSM0618**			
TT	60	8.03	**0.05**	TT	27	8.04	0.15
AA	36	7.84		CC	66	7.91	
**GSM0603**				**GSM0619**			
TT	38	8.04	0.17	TT	89	7.97	0.22
GG	57	7.92		GG	4	7.74	
**GSM0604**				**GSM0620**			
TT	68	7.97	0.46	GG	81	7.97	**0.05**
AA	26	7.96		AA	2	7.31	
**GSM0605**				**GSM0621**			
TT	24	7.66	**0.00**	AA	94	7.97	**0.05**
GG	70	8.06		TT	2	7.31	
**GSM0611**				**GSM0622**			
TT	26	7.83	0.07	GG	52	7.94	0.36
CC	67	8.03		AA	44	7.98	

**GSM0612**				**GSM0623**			
TT	37	8.06	0.10	TT	24	8.14	**0.04**
CC	55	7.91		AA	72	7.9	
**GSM0613**				**GSM0625**			
GG	39	8.05	0.13	GG	74	8.07	**0.00**
AA	53	7.92		AA	22	7.58	
**GSM0614**				**GSM0626**			
CC	40	7.98	0.39	GG	78	7.97	0.43
AA	54	7.95		AA	17	7.95	

(1) Number of genotypes classified in each allele group and (2) Average grade of the visual damage scale; nd: data not available. Values in bold highlights p-values < 0.05 obtained by t-test
