# Supplementary material for: Identification of Functional Genetic Variations Underlying Flooding Tolerance in Brazilian Soybean Genotypes
Source: Int J Mol Sci. 2022 Sep 13;23(18):10611. doi: 10.3390/ijms231810611 (PMC9502317; doi:10.3390/ijms231810611)
Supplement: Supplementary file 1 [file ijms-23-10611-s001.zip › Supplementary Table S5.pdf]

Supplementary Table S5: Associative analysis of SNPs markers and phenotyping (visual score and productivity) of soybean Brazilian genotypes submitted to flooding (Experiment 2).

Experiment 2										
**Molecular Marker**	N(1)	**1st Phenotyping**		**2nd Phenotyping**		**3rd Phenotyping**		**Productivity**	
		M(2)	*p-value*	M(3)	*p-value*	M(4)	*p-value*	M(5) (kg ha^{-1})	*p-value*
**GSM0599**									
CC	30	6.4	nd	6.08	nd	6.24	nd	1509.79	nd
GG	1	6		5		5.75		1479.2	
**GSM0600**									
AA	29	6.39	0.22	6.04	0.23	6.23	0.27	1504.48	0.22
CC	3	6.79		6.5		6.54		1285.39	
**GSM0601**									
TT	23	6.03	**0.01**	6.53	**0.01**	6.53	**0**	1357.26	**0.01**
AA	9	5.9		5.39		5.57		1807.69	
**GSM0602**									
TT	22	6.34	0.15	5.99	0.13	6.12	0.06	1591.09	**0.02**
AA	9	6.69		6.44		6.64		1222.54	
**GSM0603**									
TT	19	6.46	0.4	6.14	0.37	6.28	0.42	1477.47	0.46
GG	13	6.38		6.22		6.22		1493.41	
**GSM0604**									
TT	23	6.7	**0**	5.43	**0**	6.52	**0**	1385.58	**0.02**
AA	8	5.64		5.2		5.41		1777.87	
**GSM0605**									
TT	14	6.67	0.09	6.26	0.17	6.29	0.45	1445.02	0.28
GG	15	6.25		5.91		6.26		1542.09	
**GSM0611**									
TT	20	6.4	0.41	6.4	0.38	6.1	0.08	1566.45	0.1
CC	12	6.47		6.47		6.52		1346.43	

**GSM0612**									
TT	12	6.06	**0.03**	5.53	**0.01**	5.72	**0**	1770.12	**0**
CC	20	6.65		6.42		6.58		1312.23	
**GSM0613**									
GG	12	6.06	**0.04**	5.53	**0.01**	5.72	**0**	1770.12	**0**
AA	19	6.57		6.37		6.54		1347.16	
**GSM0614**									
CC	12	6.4	0.44	6.01	0.37	6.29	0.43	1526.35	0.35
AA	20	6.44		6.13		6.24		1458.5	
**GSM0615**									
GG	19	6.59	0.12	6.31	0.07	6.37	0.21	1415.02	0.15
AA	12	6.21		5.75		6.11		1596.94	
**GSM0616**									
TT	23	6.59	**0.04**	6.25	0.07	6.27	0.44	1433.02	0.17
CC	9	6		5.66		6.22		1614.09	
**GSM0617**									
TT	7	6.25	0.28	5.8	0.2	5.98	0.17	1576.17	0.27
AA	24	6.48		6.18		6.33		1448.16	
**GSM0618**									
TT	7	6.25	0.27	5.8	0.18	5.98	0.2	1576.17	0.27
CC	22	6.5		6.23		6.3		1445.55	
**GSM0619**									
TT	30	6.48	0.09	6.18	**0.02**	6.3	0.13	1469.78	0.26
GG	2	5.63		4.63		5.62		1696.34	
**GSM0620**									
GG	30	6.47	0.15	6.15	0.07	6.31	0.09	1472.51	0.3
AA	2	5.81		5.06		5.5		1655.39	
**GSM0621**									
AA	30	6.47	0.15	6.15	0.07	6.31	0.09	1472.51	0.3

TT	2	5.81		5.06		5.5		1655.39	
**GSM0622**									
GG	18	6.28	0.15	5.99	0.28	6.16	0.22	1565.24	0.14
AA	14	6.61		6.21		6.38		1379.42	
**GSM0623**									
TT	16	6.6	0.13	6.3	0.12	6.48	0.06	1416.24	0.21
AA	16	6.26		5.87		6.04		1551.64	
**GSM0625**									
GG	20	6.55	0.16	6.24	0.14	6.42	0.07	1378.23	**0.05**
AA	12	6.23		5.83		5.98		1660.12	
**GSM0626**									
GG	17	6.3	0.18	6.01	0.34	6.21	0.37	1524.07	0.31
AA	15	6.58		6.17		6.31		1438.47	

(1) Number of genotypes classified in each allele group; (2), (3) and (4) Mean of visual damage scales performed 8, 23 and 43 days after water drainage, respectively; (5) Average productivity; nd: data not available. Values in bold highlights p-values < 0.05 obtained by t-test.
