# Supplementary material for: Identification of Functional Genetic Variations Underlying Flooding Tolerance in Brazilian Soybean Genotypes
Source: Int J Mol Sci. 2022 Sep 13;23(18):10611. doi: 10.3390/ijms231810611 (PMC9502317; doi:10.3390/ijms231810611)
Supplement: Supplementary file 1 [file ijms-23-10611-s001.zip › Supplementary Table S6.pdf]

Supplementary Table S6: Associative analysis of SNPs markers for phenotyping (visual score) of Brazilian soybean genotypes under flooding conditions, in experiment 3.

Experiment 3									
**Molecular Marker**	N(1)	**1st Phenotyping**		**2nd Phenotyping**		**3rd Phenotyping**		**4a Phenotyping**	
		M(2)	*p-value*	M(3)	*p-value*	M(4)	*p-value*	M(5)	*p-value*
**GSM0599**									
CC	24	6.09	0.07	5.84	**0.03**	5.95	0.2	5.77	0.21
GG	2	5.5		5.06		5.56		5.32	
**GSM0600**									
AA	25	6.04	nd	6	nd	5.9	nd	5.7	nd
CC	1	6.38		6.5		6.5		6.63	
**GSM0601**									
TT	20	6.09	0.13	5.8	0.12	5.59	0.31	5.72	0.39
AA	4	5.75		5.44		5.75		5.84	
**GSM0602**									
TT	14	6.13	0.21	5.8	0.48	5.93	0.46	5.66	0.27
AA	10	5.94		5.79		5.96		5.86	
**GSM0603**									
TT	13	5.96	0.11	5.69	0.18	5.82	0.18	5.6	0.15
GG	10	6.25		5.91		6.05		5.95	
**GSM0604**									
TT	18	6.02	0.47	5.73	0.43	5.85	0.43	5.7	0.45
AA	5	6		5.78		5.9		5.65	
**GSM0605**									
TT	10	6.25	0.11	5.89	0.23	6.09	0.12	5.85	0.30
GG	13	5.96		5.71		5.79		5.67	
**GSM0611**									
TT	18	6.03	0.49	5.71	0.32	5.81	0.12	5.56	**0.02**
CC	6	6.04		5.83		6.15		6.29	

**GSM0612**									
TT	9	5.93	0.16	5.54	**0.03**	5.63	**0.02**	5.4	**0.04**
CC	15	6.17		5.98		6.16		5.99	
**GSM0613**									
GG	9	5.93	0.22	5.54	**0.04**	5.38	**0**	5.4	**0.05**
AA	15	6.12		5.95		6.13		5.95	
**GSM0614**									
CC	9	5.88	0.14	5.88	0.32	5.89	0.47	5.53	0.20
AA	16	6.13		5.79		5.91		5.8	
**GSM0615**									
GG	14	6.13	0.32	5.84	0.41	5.96	0.48	5.78	0.47
AA	10	6.01		5.79		5.95		5.75	
**GSM0616**									
TT	19	6.05	0.45	5.69	0.17	5.8	0.09	5.56	**0.03**
CC	6	6.08		5.94		6.17		6.23	
**GSM0617**									
TT	9	5.97	0.29	5.68	0.28	5.86	0.39	5.61	0.30
AA	15	6.11		5.82		5.93		5.79	
**GSM0618**									
TT	9	5.97	0.44	5.68	0.43	5.86	0.47	5.61	0.33
CC	14	5.94		5.72		5.88		5.76	
**GSM0619**									
TT	24	6.07	0.22	5.81	0.14	5.93	0.45	5.73	0.40
GG	2	5.75		5.38		5.88		5.88	
**GSM0620**									
GG	25	6.06	nd	5.83	nd	5.99	nd	5.83	nd
AA	1	5.63		4.88		5.5		5.5	
**GSM0621**									
AA	24	6.05	nd	5.81	nd	5.95	nd	5.77	nd
TT	1	5.63		4.88		5.5		5.5	
**GSM0622**									

GG	12	5.82	**0.02**	5.7	0.25	5.93	0.49	5.82	0.32
AA	14	6.24		5.85		5.92		5.69	
**GSM0623**									
TT	11	6.15	0.24	5.77	0.48	5.88	0.41	5.79	0.37
AA	13	5.98		5.76		5.93		5.68	
**GSM0625**									
GG	11	6.15	0.22	5.72	0.26	5.78	0.09	6.07	**0.03**
AA	15	5.98		5.86		6.11		5.49	
**GSM0626**									
GG	10	5.99	0.46	5.75	0.36	5.84	0.43	5.73	0.42
AA	13	6.01		5.68		5.88		5.66	

(1) Number of genotypes classified in each group and (2) (3) (4) (5) Average of visual damage scales performed 3, 15, 29, 43 and 60 days after drainage, respectively from experiment 3 in the 2018 season /19; nd: data not available. Values in bold differ by the 5% t-test for phenotyping after flooding.
