# Supplementary material for: Identification of Functional Genetic Variations Underlying Flooding Tolerance in Brazilian Soybean Genotypes
Source: Int J Mol Sci. 2022 Sep 13;23(18):10611. doi: 10.3390/ijms231810611 (PMC9502317; doi:10.3390/ijms231810611)
Supplement: Supplementary file 1 [file ijms-23-10611-s001.zip › Supplementary Table S7.pdf]

Supplementary Table S7: Associative analysis of SNPs markers for phenotyping (visual score) of Brazilian soybean genotypes under flooding conditions, in experiment 4.

Experiment 4							
Molecular Marker	N(1)	1st Phenotyping		2nd Phenotyping		3rd Phenotyping	
		M(2)	*p-value*	M(3)	*p-value*	M(4)	*p-value*
**GSM0599**							
CC	74	6.93	0.33	6.96	0.21	7.01	0.49
GG	2	6.63		6.5		7	
**GSM0600**							
AA	65	6.96	0.19	6.95	0.4	7.02	0.44
CC	11	6.68		6.89		6.98	
**GSM0601**							
TT	53	6.88	0.28	6.95	0.31	6.95	0.14
AA	22	7.02		7.02		7.17	
**GSM0602**							
TT	52	6.91	0.39	6.96	0.32	7.01	0.46
AA	17	6.84		6.85		6.99	
**GSM0603**							
TT	37	6.74	0.09	6.83	0.15	6.87	0.09
GG	36	7.05		7.03		7.13	
**GSM0604**							
TT	51	6.82	0.15	6.86	0.12	6.88	**0.03**
AA	22	7.08		7.1		7.27	
**GSM0605**							
TT	23	6.41	**0**	6.53	**0**	6.59	**0**
GG	50	7.09		7.08		7.14	
**GSM0611**							
TT	45	6.86	0.27	6.91	0.39	6.91	0.08
CC	28	7		6.96		7.18	

**GSM0612**								
TT	32	6.73	0.06	6.94	0.06	6.94	0.22
CC	41	7.09		7.09		7.09	
**GSM0613**							
GG	32	6.73	0.08	6.8	0.08	6.94	0.25
AA	39	7.06		7.06		7.07	
**GSM0614**							
CC	26	6.71	0.1	6.68	0.11	6.85	0.12
AA	49	7.01		7.02		7.08	
**GSM0615**							
GG	42	6.86	0.36	6.86	0.15	6.9	0.12
AA	29	6.94		7.03		7.13	
**GSM0616**							
TT	42	6.92	0.38	6.92	0.46	6.99	0.49
CC	31	6.85		6.94		6.99	
**GSM0617**							
TT	23	6.9	0.48	6.9	0.33	6.9	0.25
AA	50	6.89		6.86		7.04	
**GSM0618**							
TT	21	6.88	0.37	6.86	0.21	6.88	0.17
CC	52	6.96		7		7.09	
**GSM0619**							
TT	73	6.91	0.44	7	0.25	7	0.37
GG	3	7		7.25		7.17	
**GSM0620**							
GG	74	6.93	nd	6.96	nd	7.3	nd
AA	1	5.75		6		6.25	
**GSM0621**							
AA	75	6.93	nd	6.96	nd	7.28	nd
TT	1	5.75		6		6.25	
**GSM0622**							

GG	43	6.93	0.5	6.9	0.25	7.03	0.48
AA	29	6.93		7.03		7.02	
**GSM0623**							
TT	22	7.06	0.2	7.09	0.15	7.14	0.17
AA	50	7		6.88		6.94	
**GSM0625**							
GG	46	6.84	0.21	6.88	0.22	6.92	0.14
AA	28	7.03		7.03		7.13	
**GSM0626**							
GG	47	6.95	0.34	6.97	0.37	7.04	0.33
AA	29	6.86		6.91		6.96	

(1) Number of genotypes classified in each group and (2) (3) (4) Average of visual damage scales performed 5, 10, 23 and 38 days after drainage, respectively from experiment 4 in the 2018/19 season; nd: data not available. The values in bold differ by the t test at 5% regarding phenotyping after flooding
