# Supplementary material for: Identification of Functional Genetic Variations Underlying Flooding Tolerance in Brazilian Soybean Genotypes
Source: Int J Mol Sci. 2022 Sep 13;23(18):10611. doi: 10.3390/ijms231810611 (PMC9502317; doi:10.3390/ijms231810611)
Supplement: Supplementary file 1 [file ijms-23-10611-s001.zip › Supplementary Table S8.pdf]

Supplementary Table S8: Association of SNPs and productivity (kg ha^{-1}), mortality (%), NDVI and leaf retention variables in different Brazilian soybean genotypes under flooded conditions.

Experiment 3									
**Molecular Marker**	N[1]	**Productivity**		**Mortality**		**NDVI**		**Foliar Retention**	
		M[2] (kg ha^{-1})	*p-value*	M[3] (%)	*p-value*	M[4]	*p-value*	M[5]	*p-value*
**GSM0599**									
CC	24	1080.4	0.33	19.19	0.12	0.64	0.17	0.97	0.27
GG	2	1198.5		12.3		0.68		1.32	
**GSM0600**									
AA	25	1100.9	nd	18.33	nd	0.64	nd	1.01	nd
CC	1	802.4		26.8		0.59		0.5	
**GSM0601**									
TT	20	1104.5	0.45	18.99	0.3	0.65	0.39	0.93	0.23
AA	4	1080.1		16.65		0.65		1.22	
**GSM0602**									
TT	14	1156.2	0.14	20.19	0.23	0.64	0.49	0.98	0.39
AA	10	986.59		17.67		0.64		1.07	
**GSM0603**									
TT	13	1153.2	0.18	18.81	0.32	0.65	0.34	1.16	0.13
GG	10	1001.1		20.35		0.64		0.8	
**GSM0604**									
TT	18	1110.7	0.38	17.84	0.39	0.65	0.42	1	0.21
AA	5	1167.2		18.86		0.65		1.33	
**GSM0605**									
TT	10	1008.3	0.2	22.33	0.06	0.62	**0.04**	0.59	**0.01**
GG	13	1147.7		17.28		0.66		1.32	
**GSM0611**									
TT	18	1175.4	**0.04**	18.09	0.3	0.65	0.13	1.13	**0.03**
CC	6	875.42		20.12		0.63		0.5	
**GSM0612**									

TT	9	1200.8	0.11	10.22	0.21	0.66	0.09	1.18	0.13
CC	15	1003.4		12.43		0.63		0.83	
**GSM0613**									
GG	9	1200.8	0.15	10.22	0.27	0.66	0.13	1.18	0.16
AA	15	1033.4		11.97		0.63		0.85	
**GSM0614**									
CC	9	1125.9	0.4	10.33	0.34	0.65	0.43	1	0.48
AA	16	1086.8		11.41		0.65		1.02	
**GSM0615**									
GG	14	1091.1	0.42	12.18	0.31	0.64	0.34	0.82	0.15
AA	10	1058.2		10.8		0.64		1.15	
**GSM0616**									
TT	19	1151	0.11	11.05	0.36	0.65	0.13	1.04	0.49
CC	6	933.77		12.17		0.63		1.02	
**GSM0617**									
TT	9	1122.6	0.41	10.94	0.38	0.65	0.32	0.98	0.35
AA	15	1083.4		11.83		0.64		1.1	
**GSM0618**									
TT	9	1122.6	0.47	10.94	0.43	0.65	0.49	0.98	0.4
CC	14	1111		10.5		0.65		0.89	
**GSM0619**									
TT	24	1095.3	0.39	11.42	0.32	0.65	0.44	1.02	0.25
GG	2	1019		9.25		0.64		0.63	
**GSM0620**									
GG	25	1059.9	nd	19.13	nd	0.64	nd	0.99	Nd
AA	1	1045.9		13.3		0.65		0.38	
**GSM0621**									
AA	24	1092.4	nd	18.7	nd	0.64	nd	0.96	Nd
Nd	1	1045.9						0.38	
**GSM0622**									
GG	12	1127.3	0.32	10.08	0.2	0.65	0.27	1.05	0.36

AA	14	1057		12.25		0.64		0.94	
**GSM0623**									
TT	11	1112.7	0.43	12.05	0.36	0.64	0.49	0.98	0.33
AA	13	1085.8		11.04		0.64		1.12	
**GSM0625**									
GG	11	918.63	**0.02**	12.5	0.2	0.63	0.13	0.96	0.42
AA	15	1214.7		10.33		0.65		1.02	
**GSM0626**									
GG	10	1152.1	0.36	10.3	0.28	0.66	0.2	1.08	0.48
AA	13	1095.1		11.77		0.64		1.06	

(1) Number of genotypes classified in each allele group and (2) (3) (4) (5) Average productivity, mortality, NDVI and leaf retention, respectively, of soybeans in the 2018/19 season, experiment 3. nd: data not available. Values in bold highlights p-values < 0.05 obtained by t-test.
