# Supplementary figures and images for: Identification of Functional Genetic Variations Underlying Flooding Tolerance in Brazilian Soybean Genotypes
Source: Int J Mol Sci. 2022 Sep 13;23(18):10611. doi: 10.3390/ijms231810611 (PMC9502317; doi:10.3390/ijms231810611)

## TECIRGA control vs. flooding

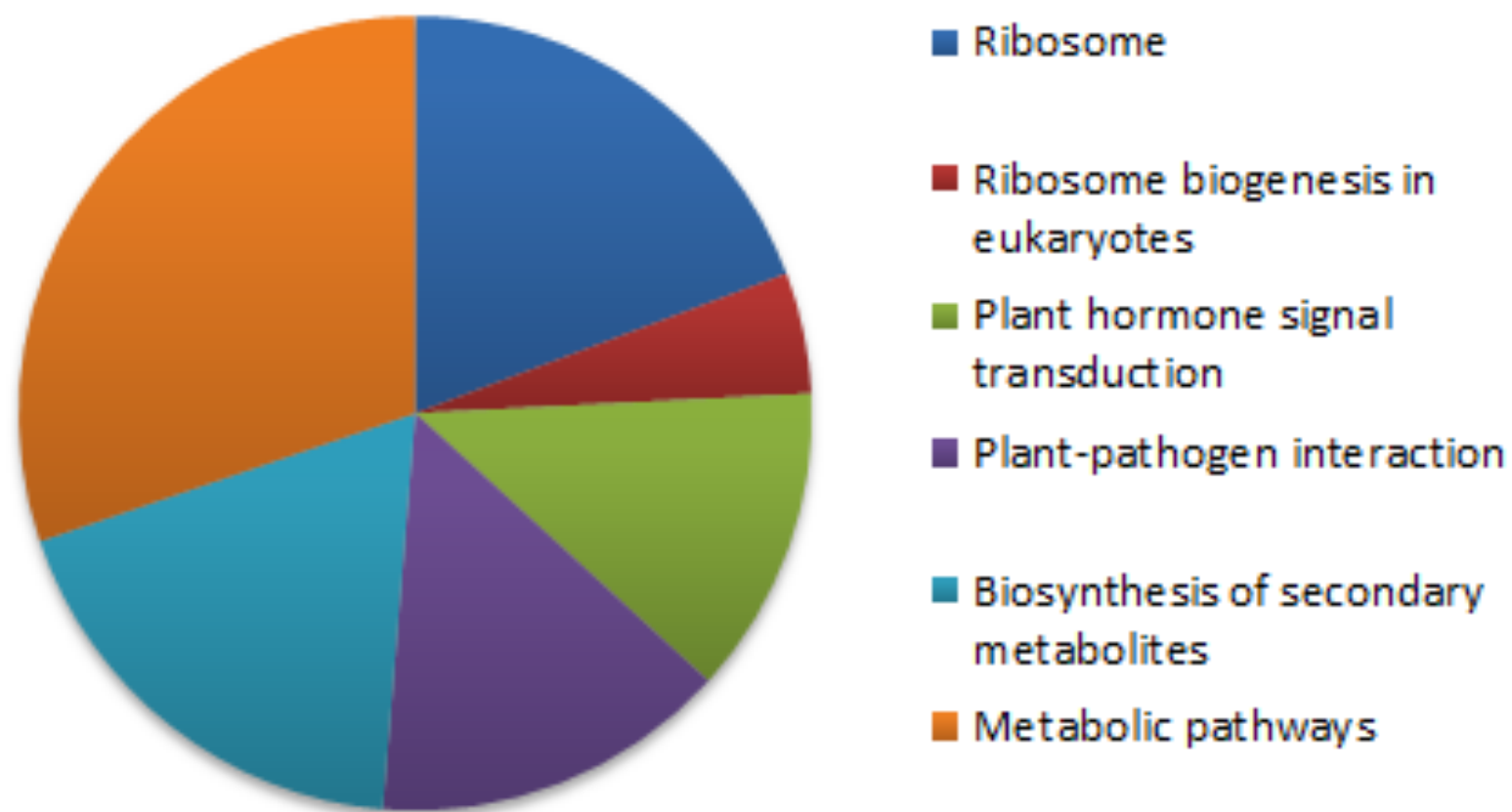

Supplement: Supplementary file 1 [file ijms-23-10611-s001.zip › Supplementary Figure S1.pdf]

## TECIRGA vs 62RR (Flooding)

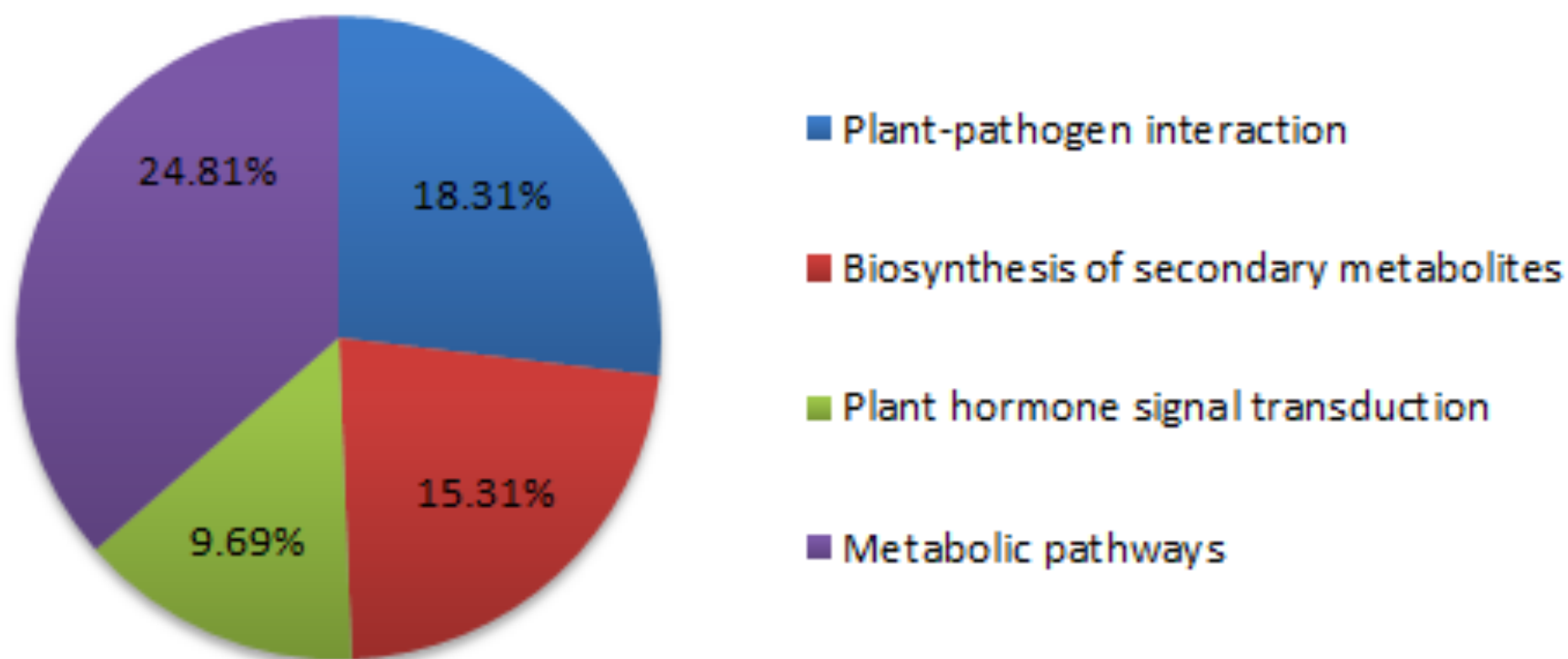

Supplement: Supplementary file 1 [file ijms-23-10611-s001.zip › Supplementary Figure S2.pdf]

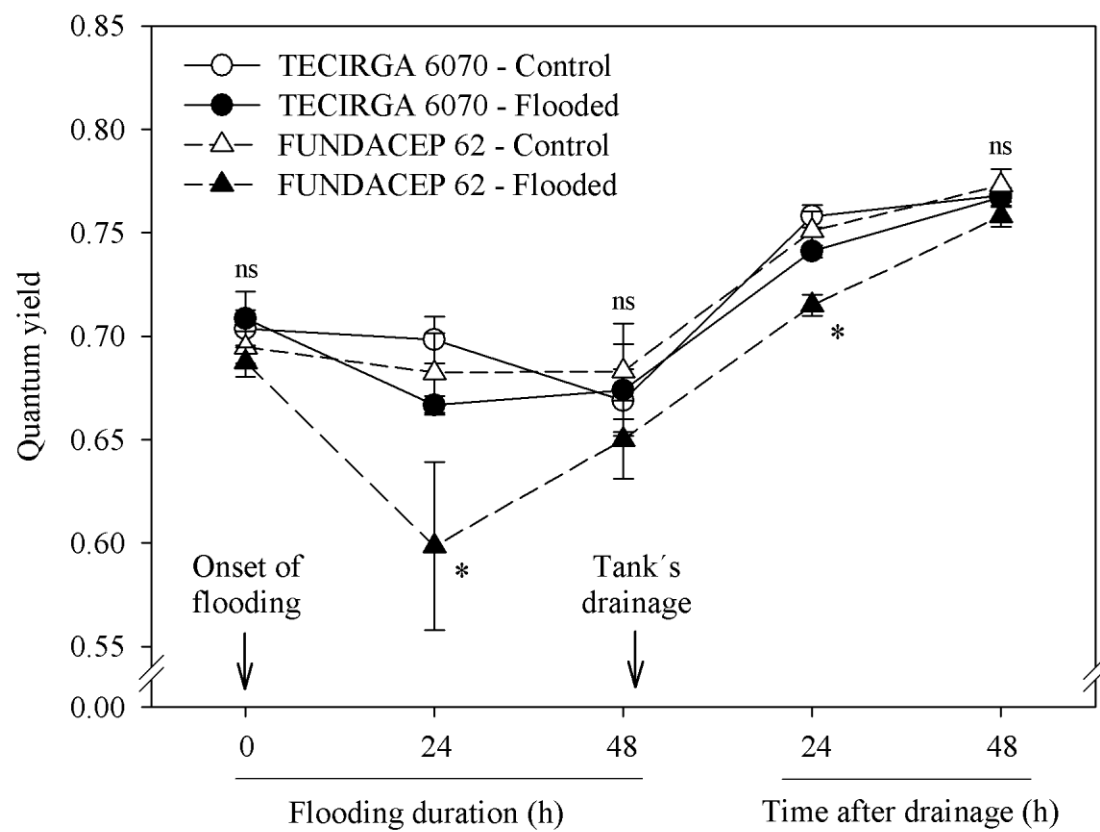

Supplement: Supplementary file 1 [file ijms-23-10611-s001.zip › Supplementary Figure S3.pdf]

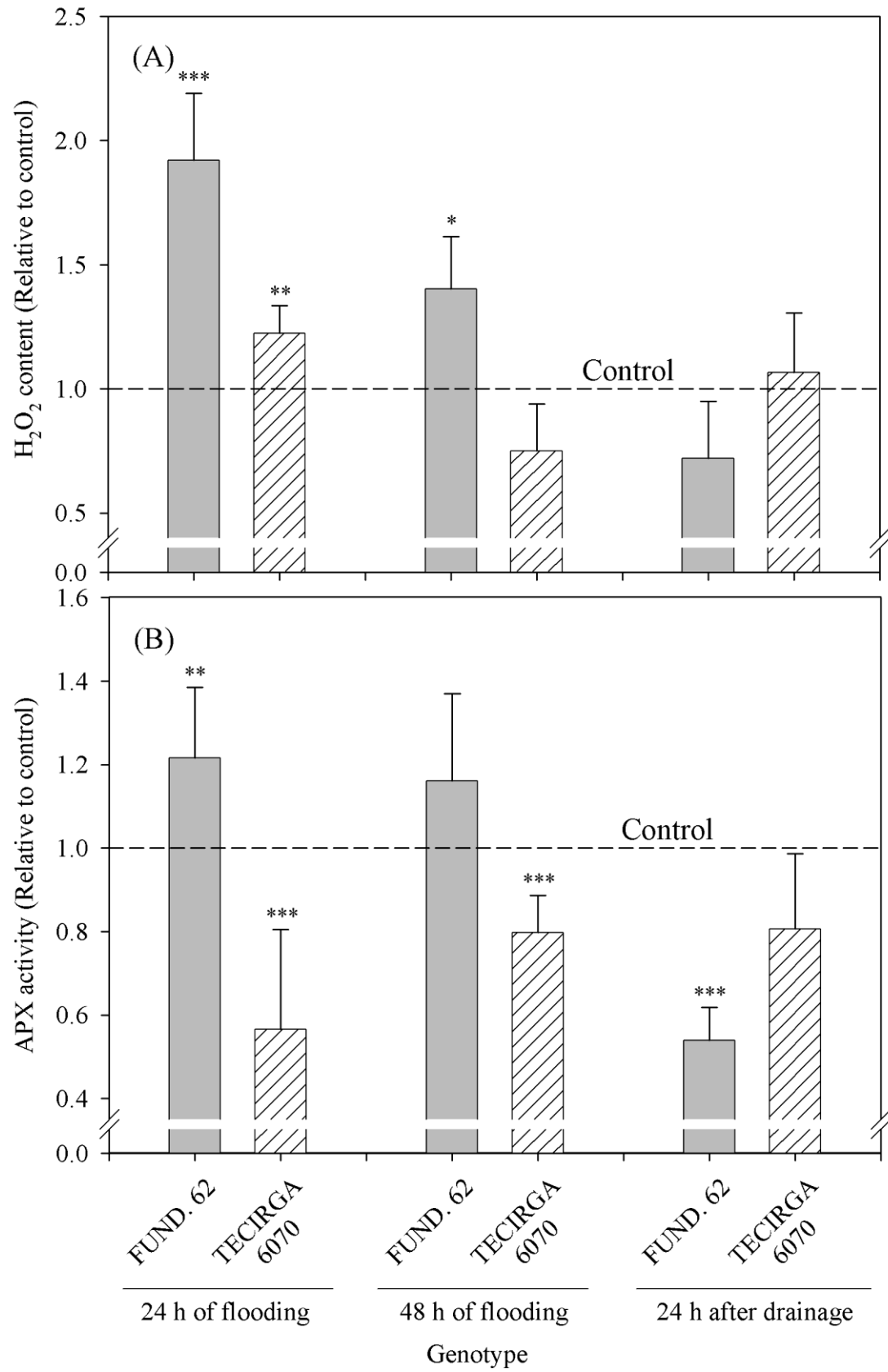

Supplement: Supplementary file 1 [file ijms-23-10611-s001.zip › Supplementary Figure S4.pdf]

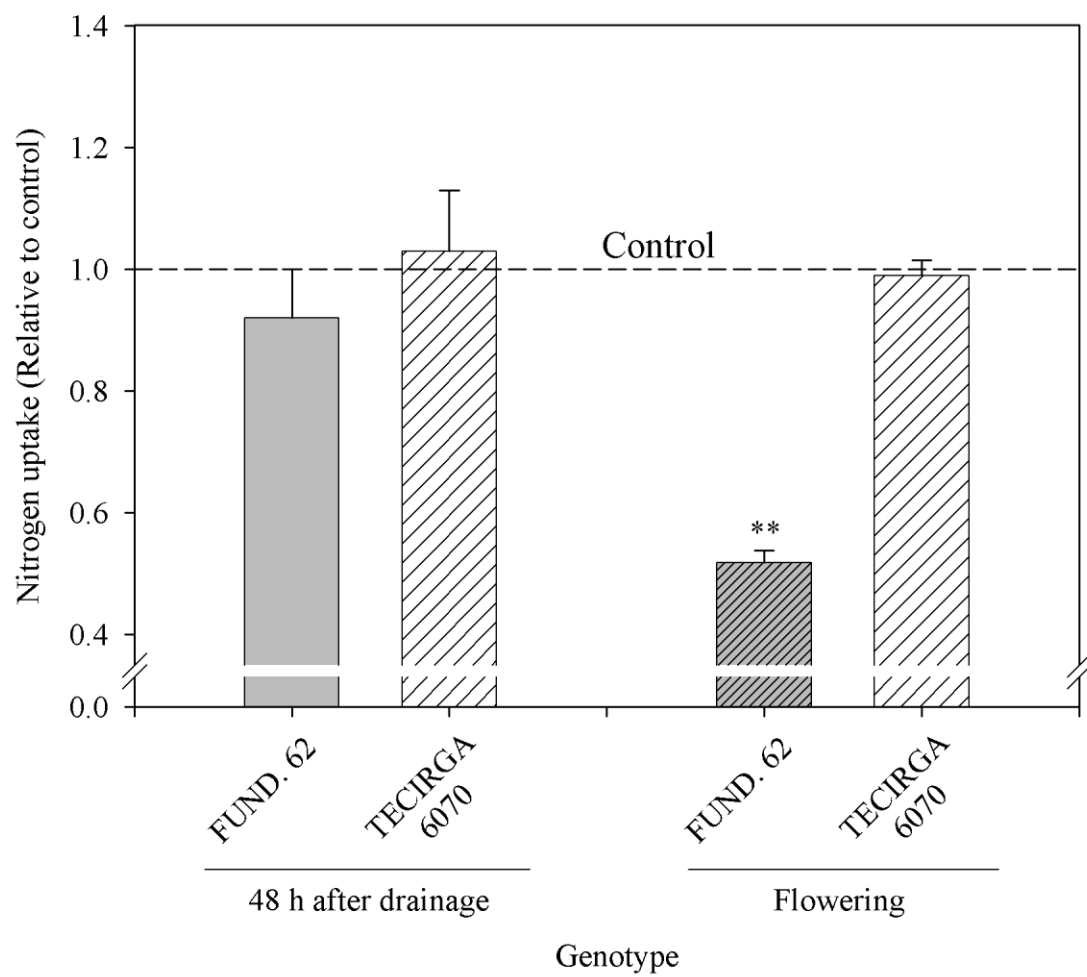

Supplement: Supplementary file 1 [file ijms-23-10611-s001.zip › Supplementary Figure S5.pdf]
